# Coupling of autophagy and the mitochondrial intrinsic apoptosis pathway modulates proteostasis and ageing in *Caenorhabditis elegans*

**DOI:** 10.1038/s41419-023-05638-x

**Published:** 2023-02-11

**Authors:** Christina Ploumi, Emmanouil Kyriakakis, Nektarios Tavernarakis

**Affiliations:** 1 Department of Basic Sciences, Faculty of Medicine, University of Crete, Heraklion, 71003 Crete Greece; 2 Institute of Molecular Biology and Biotechnology, Foundation for Research and Technology-Hellas, Heraklion, 70013 Crete Greece; 3 Biozentrum, University of Basel, Basel, Switzerland

**Keywords:** Apoptosis, Macroautophagy, Mitochondria

## Abstract

Mitochondria preserve metabolic homeostasis and integrate stress signals, to trigger cytoprotective, or cell death pathways. Mitochondrial homeostasis and function decline with age. The mechanisms underlying the deterioration of mitochondrial homeostasis during ageing, or in age-associated pathologies, remain unclear. Here, we show that CISD-1, a mitochondrial iron-sulfur cluster binding protein, implicated in the pathogenesis of Wolfram neurodegenerative syndrome type 2, modulates longevity in the nematode *Caenorhabditis elegans* by engaging autophagy and the mitochondrial intrinsic apoptosis pathway. The anti-apoptotic protein CED-9 is the downstream effector that mediates CISD-1-dependent effects on proteostasis, neuronal integrity and lifespan. Moreover, intracellular iron abundance is critical for CISD-1 function, since mild iron supplementation is sufficient to decelerate ageing and partly ameliorate the disturbed mitochondrial bioenergetics and proteostasis of CISD-1 deficient animals. Our findings reveal that CISD-1 serves as a mechanistic link between autophagy and the apoptotic pathway in mitochondria to differentially modulate organismal proteostasis and ageing, and suggest novel approaches which could facilitate the treatment of Wolfram Syndrome or related diseases.

## Introduction

Ageing is a universal phenomenon, initiated by the progressive functional decline of cellular processes, gradually leading to systemic and organismal failure. Among the major hallmarks of ageing and age-related pathologies is mitochondrial dysfunction [1]. Increased generation of mitochondria-derived reactive oxygen species (ROS) [2], accumulation of mutations in mitochondrial DNA [3], as well as deregulation of the mitochondrial unfolded protein response (UPR^mt^) [4, 5], are some of the mechanisms suggested to contribute to the ageing process and the development of age-associated maladies.

While ample evidence supports that mitochondrial function is a key determinant of lifespan, recent studies have re-evaluated its implication in ageing. For example, ROS can act as signal transducers, activating downstream mechanisms, including autophagy and the intrinsic (or mitochondria-derived) apoptosis pathway, to alleviate stress-induced damage [6–8]. Autophagy acts as a first line of defense, by removing toxic protein aggregates and dysfunctional organelles including mitochondria. Deregulation or inability of the autophagic process to mitigate stress leads to the activation of pro-apoptotic mechanisms which mediate the elimination of stressed cells [9]. Intriguingly, in certain vertebrate tissues, as well as in the nematode *Caenorhabditis elegans*, stimulation of the intrinsic apoptosis pathway acts as a pro-longevity cue, triggering stress resistance mechanisms rather than cell death [10]. During ageing, autophagic [11, 12] and apoptotic [13] signaling pathways decline or become inoperative, ultimately leading to detrimental effects for the aged organism. Mitochondria are considered to be the main hubs, where autophagic and apoptotic machineries converge [14]. However, the mitochondrial factors that orchestrate the cross-talk between autophagy and apoptosis as well as the exact mechanisms through which they regulate longevity and healthspan remain largely unexplored.

The NEET family of iron–sulfur (Fe–S) cluster-binding proteins is characterized by the presence of the conserved CDGSH domain. All three mammalian CDGSH Iron Sulfur Domain containing (CISD) proteins are predominantly localized in mitochondria. CISD1/mitoNEET (mitoNEET for mitochondrial NEET) and CISD2/NAF-1 (NAF-1 for nutrient-deprivation autophagy factor-1), both harbor one CDGSH domain, and reside in the outer mitochondrial membrane (OMM), as homodimers, while the third member CISD3/MiNT (mitochondrial inner NEET protein), that contains two CDGSH domains resides in mitochondrial matrix, in a monomeric form. Several studies have pointed out the anti-apoptotic role of NEETs [15, 16]. Notably, all CISD proteins have been implicated in the regulation of apoptosis that occurs physiologically in the germline of *C. elegans* [17]. CISD2/NAF-1, which also localizes in the endoplasmic reticulum (ER), modulates the autophagic pathway. Particularly, as shown in human non-small cell carcinoma cell lines, CISD2/NAF-1 interacts with the anti-apoptotic protein B-Cell CLL/Lymphoma 2 (BCL2) and this interaction is required for the BCL2–dependent inhibition of the autophagosome initiating complex [18, 19]. Mutations in CISD2/NAF-1 have been linked with Wolfram syndrome type 2 (WS2), a rare, devastating neurodegenerative disorder [20]. WS2 patients suffer from juvenile diabetes mellitus, early optic atrophy and deafness, psychiatric disorders and have a significantly reduced life expectancy [21]. In accordance, *cisd2*^*-/-*^ mice are significantly short-lived and display features of premature ageing [22]. Conversely, CISD2 overexpression promotes longevity and abrogates muscle and neuronal degeneration in mice [23]. However, the mechanisms mediating the CISD2 pro-longevity effects remain unclear.

We hypothesized that mitochondrially localized CISDs modulate autophagic and apoptotic pathways in response to stress, through their redox modulatory capacity associated with binding to Fe/S clusters. To investigate this hypothesis, we focused on CISD-1, the single ortholog of CISD1/MitoNEET and CISD2/NAF-1 in *C. elegans*. We found that CISD-1 deficient nematodes display common features associated with NEET protein deficiency in mammals, including reduced lifespan and pronounced neurodegeneration. Additionally, *cisd-1* silencing perturbs cellular proteostasis in nematode models of Parkinson’s and Huntington’s diseases. Our results indicate that the intrinsic apoptosis pathway mediates the pro-longevity effects of CISD-1. Notably, the detrimental consequences of CISD-1 deficiency in lifespan, neuronal integrity and proteostasis are fully rescued upon depletion of the anti-apoptotic protein CED-9, through induction of autophagy. CISD-1 differentially affects longevity and healthspan, in a CED-9 dependent manner, engaging both the apoptotic and autophagic pathways. Moreover, CISD-1 modulates longevity in a manner that depends on mitochondrial function and intracellular iron abundance. Our work identifies CISD-1 as a mitochondrial effector that couples autophagy and the apoptosis pathway to modulate organismal healthspan and longevity.

## Results

### CISD-1 deficiency recapitulates Wolfram syndrome type 2 traits

**Fig. 1:**
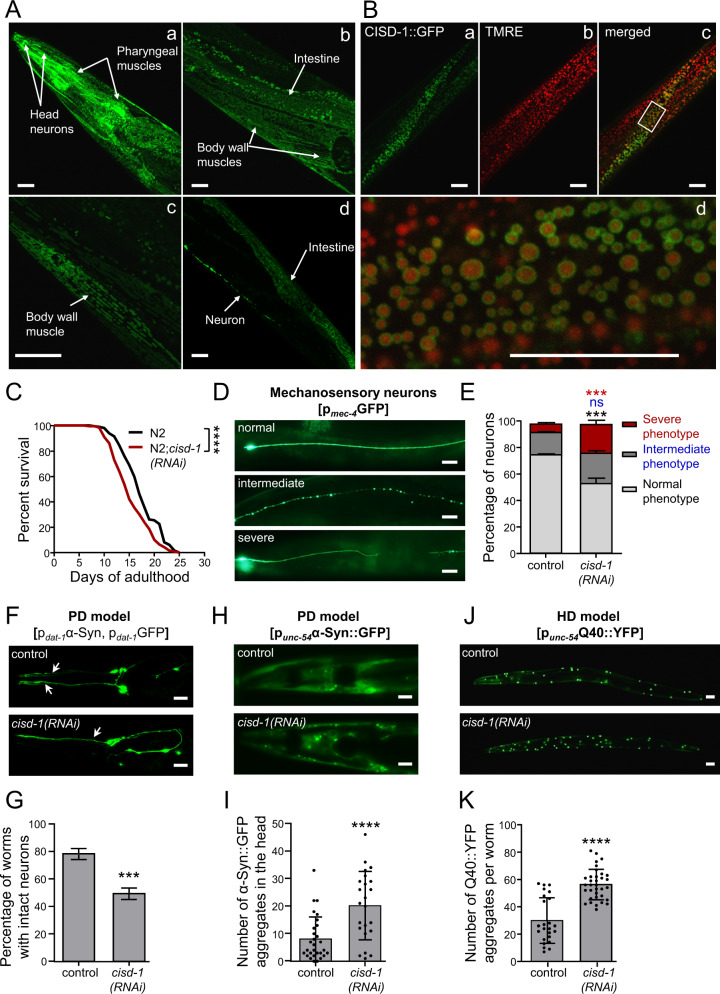
CISD-1 is a ubiquitously expressed OMM protein which preserves longevity, proteostasis, and neuronal integrity. **A** Confocal imaging of day-2 adult p_*CEOP2540*_CISD-1::GFP transgenic animals. **B** Confocal imaging of day-2 adult p_*CEOP2540*_CISD-1::GFP transgenic animals, stained with TMRE. **C** Survival curves of wt (N2) animals treated with empty vector (black line) or *cisd-1(RNAi)* (red line) from hatching (****P < 0.0001, Log-rank/Mantel–Cox test). **D** Mechanosensory neurons were observed by using day-4 adult animals of the SK4005 [p_*mec-4*_GFP] reporter strain, treated with empty vector (control) or *cisd-1(RNAi)*. The neurons were classified into three categories depending on the axonal morphology (normal, intermediate and severe phenotype). Representative images of each category are shown. **E** Quantification of the percentage of mechanosensory neurons with normal, intermediate or severe phenotype. Values represent mean ± SEM from three independent experiments (*n* = 40; ns *P* > 0.05, ***P < 0.001, two-way ANOVA/ Sidak’s multiple comparison test). **F** Representative images of head dopaminergic neurons in day-3 animals of the PD model strain UA196 [*sid-1(pk3321)*;*baIn33* (p_*dat-1*_SID-1, p_*myo-2*_mCherry); (p_*dat-1*_α-Syn, p_*dat-1*_GFP)], treated with empty vector (control) or *cisd-1(RNAi)*. Arrowheads indicate intact neurons. **G** Quantification of the percentage of dopaminergic neurons that remain intact in the heads of RNAi treated UA196 nematodes. Values represent mean ± SEM from five independent experiments (*n* = 20; ***P < 0.001, unpaired t-test). **H** Representative images of head body wall muscles in day-4 animals of the PD model strain UA49 [p_*unc-54*_α-Syn::GFP], treated with empty vector (control) or *cisd-1(RNAi)*. **I** Quantification analysis of α-Syn::GFP puncta in body wall muscles of the head region. Values represent mean ± SD (*n* = 35; ****P < 0.0001, unpaired t-test). **J** Representative images of day-1 adult animals of the HD model strain AM141 [p_*unc-54*_Q40::YFP] treated with empty vector (control) or *cisd-1(RNAi)*. **K** Quantification analysis of Q40::YFP puncta in the entire body. Values represent mean ± SD (*n* = 35; ****P < 0.0001, unpaired t-test). Scale bars in all panels are 20μm.

To investigate the physiological function and subcellular localization of CISD-1 in *C. elegans*, we generated translational reporter lines expressing the green fluorescent protein (GFP) fused to the C-terminus of CISD-1. The expression of the transgene is driven by the promoter of CEOP2540 operon, on which *cisd-1* gene is embedded. CISD-1 is ubiquitously expressed throughout the animal body, particularly in body wall muscles, neurons and intestinal cells, displaying a unique subcellular expression pattern reminiscent of mitochondria-localized proteins (Fig. 1A). To verify its mitochondrial localization we stained the p_*CEOP2540*_CISD-1::GFP transgenic animals with TMRE (TetraMethyl Rhodamine Ethyl Ester), a red fluorescent dye, which selectively accumulates in the matrix of functional mitochondria in a membrane potential-dependent manner [24]. CISD-1::GFP encircles TMRE-stained mitochondrial matrices, supporting that CISD-1 is an OMM protein, as previously reported [25] (Fig. 1B). To our knowledge, this is the only iron–sulfur-containing protein present on the OMM of *C. elegans*.

To examine its role in pathophysiology, we silenced *cisd-1* by RNAi feeding. The efficacy of the RNAi treatment was validated by quantitative reverse transcription PCR (RT-qPCR) and epifluorescence microscopy. RT-qPCR analysis revealed that feeding with *cisd-1(RNAi)*-producing bacteria significantly reduces the expression of the endogenous *cisd-1* gene (Fig. S1A). Accordingly, protein expression evaluated by measuring the mean fluorescence intensity of p_*CEOP2540*_CISD-1::GFP transgenic worms is significantly lower upon *cisd-1(RNAi)*, indicating effective silencing (Fig. S1B, C). To assess the role of CISD-1 in longevity, we performed survival assays upon *cisd-1(RNAi)* in wild-type animals (N2). In line with mammalian studies, *cisd-1* downregulation significantly shortens the lifespan of wild-type nematodes (Fig. 1C). Nevertheless, a recent study conducted in *C. elegans* reported that the null *cisd-1(tm4993)* mutants display normal longevity [25]. To resolve this discrepancy, we used the same mutants after outcrossing them four times with a wild-type (wt) strain. The outcrossed *cisd-1(tm4993)* mutants display significantly reduced mean lifespan compared to the wt (N2) and the initial, not-outcrossed, *cisd-1(tm4993)* strain (Fig. S2A), supporting our initial RNAi experiments.

We then investigated whether CISD-1 deficiency affects neuronal integrity. We used the SK4005 [p_*mec-4*_GFP] strain to assess axonal morphology of mechanosensory neurons, which are RNAi-sensitive [26]. We classified the neurons into three categories, depending on their axonal morphology: severe phenotype (completely disrupted, with breaks, branches and fork-like structures), intermediate phenotype (thin and “beaded” axons) and normal phenotype (Fig. 1D). We found that *cisd-1(RNAi)*-treated animals display a significantly higher percentage of severely-affected neurons (Fig. 1E). Similar defects were observed in the mechanosensory neurons of the outcrossed *cisd-1(tm4993)* mutants (Fig. S2B). We also evaluated whether *cisd-1* downregulation accelerates neurodegeneration by using the aggregation-prone nematode model for Parkinson’s disease (PD), UA196 [*sid-1(pk3321)*; *baIn33*(p_*dat-1*_SID-1, p_*myo-2*_mCherry);(p_*dat-1*_α-Syn, p_*dat-1*_GFP)]. This is a neuronal RNAi sensitive strain which expresses the human alpha-synuclein (α-Syn) protein in dopaminergic neurons. We observed that nematodes subjected to *cisd-1(RNAi)* display accelerated dopaminergic neuronal loss compared to their control counterparts (Fig. 1F, G).

Loss of proteostasis, is a main hallmark of ageing, and promotes the accumulation of protein aggregates, which are commonly associated with the manifestation of age-related pathologies, such as neurodegenerative disorders [1]. Thus, we hypothesized that loss of CISD-1 perturbs proteostasis, which may, in turn, aggravate neuronal degeneration. To assess perturbation of proteostasis we utilized two nematode strains expressing the PD-associated protein α-Syn fused to GFP [p_*unc-54*_α-Syn::GFP] and the Huntington’s Disease (HD)-associated segment of polyglutamine (Q40) repeats, fused to YFP [p_*unc-54*_Q40::YFP] respectively, in their body wall muscles. We found that cellular proteostasis is impaired upon *cisd-1* silencing, since the number of α-Syn::GFP and Q40::YFP aggregates are significantly elevated in *cisd-1(RNAi)*-treated animals, compared to the respective controls (Fig. 1H–K). Indeed, *cisd-1* ablation markedly enhances α-Syn::GFP and Q40::YFP aggregation (Fig.S2C–F). While, muscle degeneration is evident in *Cisd2* KO mice as a result of disturbed mitochondrial homeostasis [22], we did not observe significant changes in either muscular morphology, or the muscle mitochondrial network (Fig. S3A, B). Nevertheless, CISD-1 deficient nematodes display reduced body bends, indicating locomotory defects (Fig. S3C). These defects may arise from compromised proteostasis and enhanced neurodegeneration in CISD-1 deficient animals. Overall, the aforementioned findings suggest that CISD-1 safeguards proteostasis, neuronal integrity, health- and lifespan. Thus, CISD-1 depletion in *C. elegans* recapitulates in vitro and in vivo findings in mammalian models, and induces features associated with mitochondria-related disorders, such as neurodegenerative diseases and WS2, supporting an evolutionary conserved role of CISD proteins in preserving animal physiology.

### CISD-1 differentially regulates longevity, contingent on mitochondrial activity

**Fig. 2:**
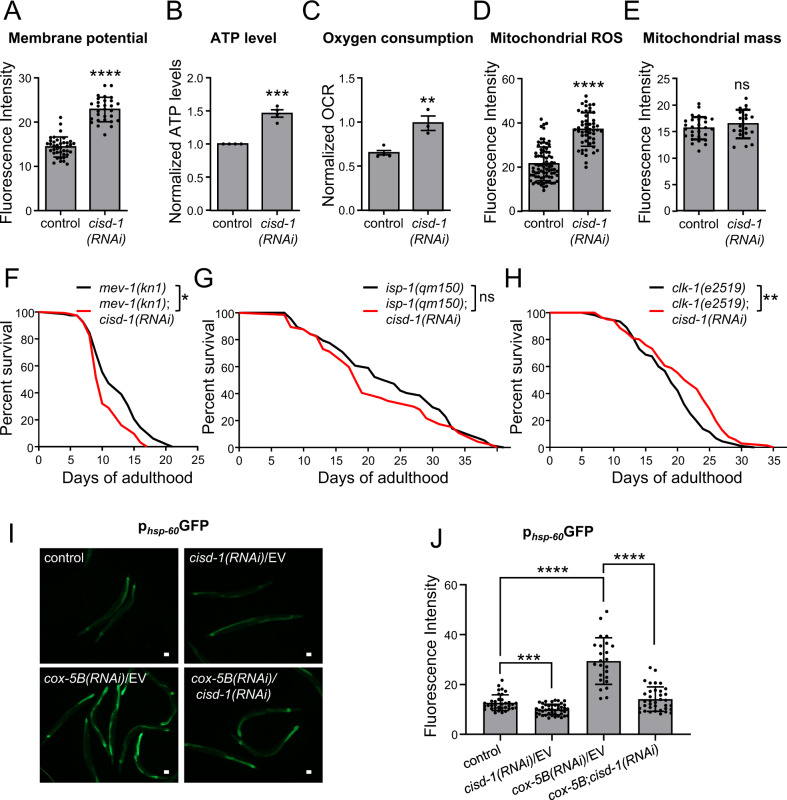
CISD-1 regulates mitochondrial bioenergetics, and its pro-longevity effects require intact mitochondrial function. **A**. Membrane potential of day-1 wt (N2) animals, treated with either empty vector (control) or *cisd-1(RNAi)* and supplemented overnight with the selective mitochondrial dye TMRE. Values represent mean ± SD (*n* = 30; ****P < 0.0001, unpaired t-test). **B** ATP levels were determined in day-1 wt (N2) animals treated with either empty vector (control) or *cisd-1(RNAi)*. The graph summarizes the results of four independent experiments and ATP levels are shown relative to control samples (wt animals treated with empty vector). Values represent mean ± SEM (***P < 0.001, unpaired t-test). **C** Oxygen consumption rate (OCR) was determined in day-1 wt (N2) animals, treated with either empty vector (control) or *cisd-1(RNAi)*. One representative experiment is shown, with four technical replicates per condition. OCR was normalized to total protein levels. Values represent mean ± SD (**P < 0.01, unpaired t-test). **D** For the determination of mitochondrial ROS, mean fluorescence intensity was measured in day-1 wt (N2) animals treated either with empty vector (control) or *cisd-1(RNAi)*. L4-staged animals were supplemented overnight with the selective mitochondrial dye MitoTracker™ Red CM-H_2_Xros. Values represent mean ± SD (*n* = 50; ****P < 0.0001, unpaired t-test). **E** Mitochondrial mass of day-1 adult nematodes of the SJ4143 [p_*ges-1*_mtGFP] strain, treated with either empty vector (control) or *cisd-1(RNAi)*. Values represent mean ± SD (*n* = 25; ns *P* > 0.05, unpaired t-test). **F** Survival curves of *mev-1(kn1)* animals treated with empty vector (black line) or *cisd-1(RNAi)* (red line) from hatching (*P < 0.05, Log-rank/Mantel–Cox test). **G** Survival curves of *isp-1(qm150)* animals treated with empty vector (black line) or *cisd-1(RNAi)* (red line) from hatching (ns, *P* > 0.05, Log-rank/Mantel–Cox test). **H** Survival curves of *clk-1(e2519)* animals treated with empty vector (black line) or *cisd-1(RNAi)* (red line) from hatching (**P < 0.01, Log-rank/Mantel–Cox test). I. Representative images of day-2 animals of the UPR^mt^ reporter strain SJ4058 [p_*hsp-60*_GFP], treated with empty vector (control), *cisd-1(RNAi)* (diluted with empty vector), *cox-5B(RNAi)* (diluted with empty vector) and double RNAi for *cisd-1* and *cox-5B*. **J** Quantification of the mean fluorescence intensity of RNAi-treated animals of the SJ4058 strain. Values represent mean ± SD (*n* = 35; ***P < 0.001, ****P < 0.0001, one-way ANOVA/ Tamhane’s T2 multiple comparisons test). Scale bars in all panels are 20 μm.

We, next, examined the role of CISD-1 in mitochondrial function and homeostasis, given its localization at the OMM. We found that *cisd-1* silencing significantly increases mitochondrial membrane potential, ATP levels, basal oxygen consumption rate (OCR), and mitochondrial ROS, while mitochondrial mass remains unaffected (Fig. 2A–E). These findings indicate that CISD-1 is essential for sustaining mitochondrial bioenergetics.

Dysfunctional mitochondria have been associated with the ageing process and the development of age-associated diseases [1]. Hence, we investigated the role of key electron transport chain (ETC) proteins in CISD-1-dependent longevity. Disruption of mitochondrial function in *C. elegans* leads to severe consequences, such as cell death and organismal senescence. Paradoxically, hypomorphic mutations in specific ETC components lead to lifespan extension [27–29]. We found that *cisd-1* silencing in the short-lived *mev-1(kn1)* mutants (*mev-1* encodes the succinate dehydrogenase cytochrome b560 subunit in complex II of ETC) shortens lifespan similarly to its effect on wt animals (Fig. 2F). In contrast, the lifespan of long-lived *isp-1(qm150)* (*isp-1* encodes the Rieske iron sulfur protein subunit of cytochrome c oxidoreductase, found in complex III) and *clk-1(e2519)* (*clk-1* encodes the coenzyme Q, hydroxylase) mutant animals upon *cisd-1(RNAi)* remains unaffected (Fig. 2G), or is mildly extended (Fig. 2H), respectively. Thus, disruption of ETC differentially affects the lifespan of CISD-1 deficient nematodes.

Perturbation of ETC function activates the mitochondrial unfolded protein response (UPR^mt^), a stress defense mechanism, required for ETC perturbation-mediated longevity [30, 31]. Downregulation of *cisd-1* curtails UPR^mt^, as indicated by the reduced expression of the SJ4058 [p_*hsp-60*_GFP] transcriptional reporter, under both normal and *cox-5B(RNAi)* conditions (Fig. 2I, J). The *cox-5B* gene encodes the cytochrome c oxidase subunit 5B of ETC complex IV, and its inhibition potently induces UPR^mt^. These findings suggest that CISD-1 regulates UPR^mt^ upstream of COX-5B. Thus, alterations in ETC function combined with UPR^mt^ downregulation likely underlies the shortening of *cisd-1(RNAi)*-treated animal lifespan. To further investigate whether CISD-1 is involved in other stress response mechanisms, we measured the expression of *hsp-4* and *hsp-16.2* genes by using the transcriptional reporters SJ4005 [p_*hsp-4*_GFP] (Fig. S4A, B) and CL2070 [p_*hsp-16.2*_GFP] (Fig. S4C, D) respectively. HSP-4 is the nematode’s ortholog for the ER chaperone GRP78-BiP, which is upregulated upon induction of the ER unfolded protein response (UPR^ER^), while HSP-16.2 is the ortholog for human crystalline alpha B protein, which is transcriptionally induced when the Heat-Shock response (HSR) pathway is activated. Silencing of *cisd-1* does not significantly alter UPR^ER^ or HSR under normal conditions. Interestingly, upon UPR^ER^-inducing conditions (*tunicamycin* treatment) the *cisd-1(RNAi)* treated worms display increased *hsp-4* activity compared to the controls. Given that CISD-1 deficient animals display impaired proteostasis (Fig. 1H–K), an additional stressor could further induce UPR^ER^. Thus, we conclude that CISD-1 is specifically linked with the mitochondrial unfolded protein response and not the cognate ER or cytosolic-associated defensive mechanisms, at least under physiological conditions. Combined, our findings suggest that CISD-1-dependent effects on lifespan are associated to ETC and mitochondrial function.

### The intrinsic apoptosis pathway mediates CISD-1 effects on ageing

**Fig. 3:**
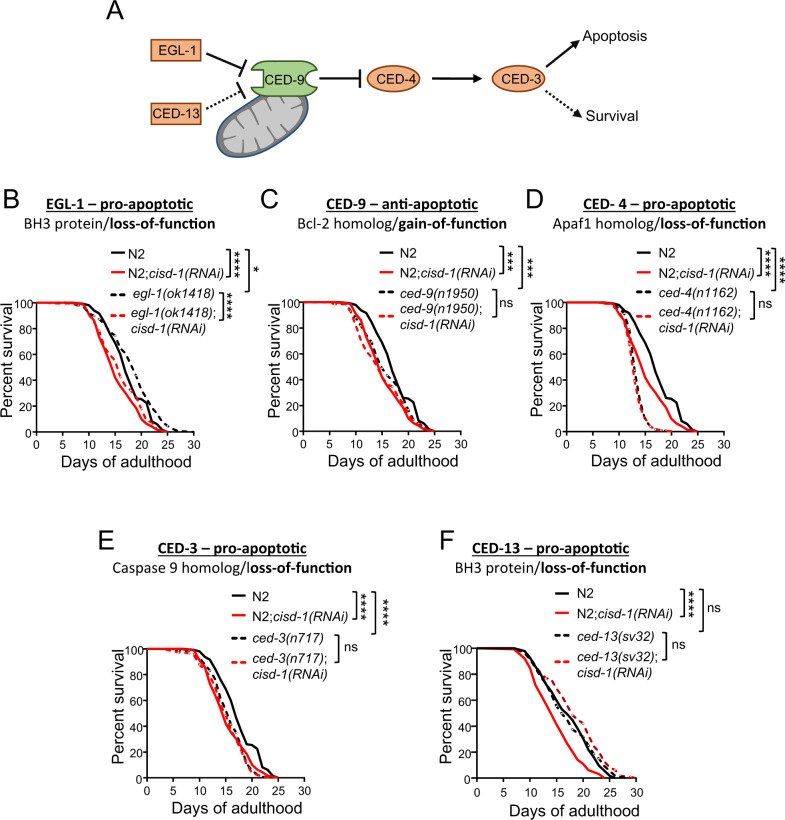
The intrinsic apoptosis pathway mediates CISD-1 pro-longevity effects. **A** Representation of the *C. elegans* apoptotic pathway. **B** Survival curves of wt (normal solid line) and *egl-1(ok1418)* (dashed line), treated with either empty vector (black line) or *cisd-1(RNAi)* (red line) from hatching (*P < 0.05, ****P < 0.0001, Log-rank/Mantel–Cox test). **C** Survival curves of wt (normal solid line) and *ced-9(n1950)* (dashed line), treated with either empty vector (black line) or *cisd-1(RNAi)* (red line) from hatching (ns *P* > 0.05, ***P < 0.001, Log-rank/Mantel-Cox test). **D** Survival curves of wt (normal solid line) and *ced-4(n1162)* (dashed line), treated with either empty vector (black line) or *cisd-1(RNAi)* (red line) from hatching (ns *P* > 0.05, ****P < 0.0001, Log-rank/Mantel–Cox test). **E** Survival curves of wt (normal solid line) and *ced-3(n717)* (dashed line), treated with either empty vector (black line) or *cisd-1(RNAi)* (red line) from hatching (ns *P* > 0.05, ****P < 0.0001, Log-rank/Mantel–Cox test). **F** Survival curves of wt (normal solid line) and *ced-13(sv32)* (dashed line), treated with either empty vector (black line) or *cisd-1(RNAi)* (red line) from hatching (ns *P* > 0.05, ****P < 0.0001, Log-rank/Mantel–Cox test).

Apoptosis has previously been implicated in cellular responses linking mitochondrial activity and ageing [10, 32]. In consistence with a recent study reporting an anti-apoptotic role of CISD-1 in *C. elegans* [17], we found that *cisd-1* lesion induces apoptosis, as manifested by the increased number of CED-1-positive apoptotic corpses in the nematode gonads (Fig. S5A, B). Next, we assessed the involvement of the mitochondrially-derived, intrinsic apoptosis pathway on CISD-1-dependent longevity. The canonical pathway involves four main factors, the pro-apoptotic BH3 (BCL2 homology domain 3)-only protein EGL-1, the anti-apoptotic BCL2 ortholog protein CED-9, the pro-apoptotic APAF1 ortholog protein CED-4 and the caspase 9 ortholog protein CED-3. CED-9 is tethered to the OMM, where it binds and inhibits CED-4. Binding of EGL-1 to CED-9 changes its conformation, allowing CED-4 to activate the executor caspase CED-3. CED-13 is an alternative BH3 negative regulator of CED-9, and the CED-13 apoptotic pathway has been suggested to promote survival instead of death by sensing disturbances in mitochondrial function [7, 10] (Fig. 3A). To examine whether cell death plays a role in CISD-1-dependent effects on longevity, we performed lifespan assays with intrinsic apoptosis pathway mutants. Downregulation of *cisd-1* in the loss-of-function *egl-1(ok1418)* mutants shortens lifespan similarly to wt nematodes (Fig. 3B), suggesting that CISD-1 acts downstream or independently of EGL-1. In contrast, *cisd-1(RNAi)* does not further shorten the lifespan of gain-of-function *ced-9(n1950)* mutants (Fig. 3C). The same outcome is observed in the loss-of-function *ced-4(n1162)* and *ced-3(n717)* mutants (Fig. 3D, E), indicating that CISD-1, CED-4, and CED-9 act through the same pathway to regulate longevity. Next, we addressed the involvement of the alternative CED-13 pathway by using the loss-of-function mutants *ced-13(sv32)*. Remarkably, in a similar manner, *cisd-1(RNAi)* does not lead to lifespan shortening in *ced-13(sv32)* mutants (Fig. 3F), supporting the involvement of the alternative CED-13-apoptosis pathway in CISD-1 mediated longevity. Altogether, these findings suggest that the deleterious effects of CISD-1 depletion on nematode’s survival, are mediated by the alternative intrinsic apoptosis pathway, independently of EGL-1.

### CED-9 functions downstream of CISD-1 to modulate lifespan, through autophagy

**Fig. 4:**
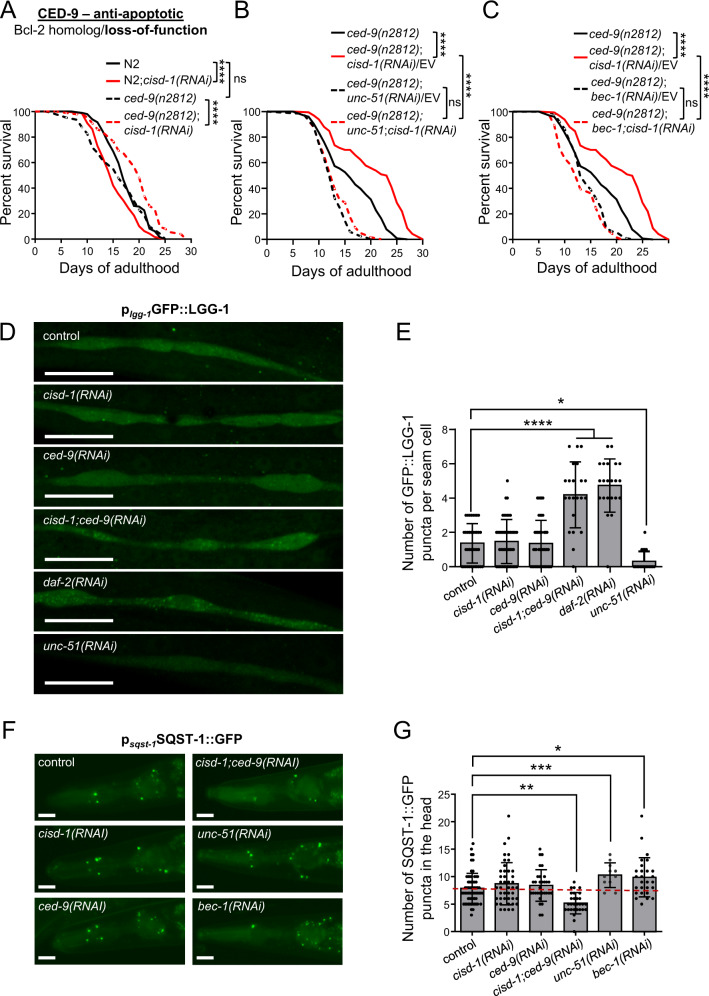
CED-9 functions downstream of CISD-1 to modulate longevity through autophagy. **A** Survival curves of wt (normal solid line) and *ced-9(n2812)* (dashed line), treated with either empty vector (black line) or *cisd-1(RNAi)* (red line) from hatching (ns *P* > 0.05, ****P < 0.0001, Log-rank/Mantel–Cox test). **B** Survival curves of *ced-9(n2812)* animals treated with empty vector (normal black line), *cisd-1(RNAi)* diluted with empty vector (normal red line), *unc-51(RNAi)* diluted with empty vector (black dashed line) and double RNAi for *cisd-1* and *unc-51* genes (red dashed line) from hatching (ns *P* > 0.05, ****P < 0.0001, Log-rank/Mantel–Cox test). **C** Survival curves of *ced-9(n2812)* animals treated with empty vector (normal black line), *cisd-1(RNAi)* diluted with empty vector (normal red line), *bec-1(RNAi)* diluted with empty vector (black dashed line) and double RNAi for *cisd-1* and *bec-1* genes (red dashed line) from hatching (ns *P* > 0.05, ****P < 0.0001, Log-rank/Mantel–Cox test). **D** Representative images of hypodermal seam cells, in L4 animals of the DA2123 [p_*lgg-1*_GFP::LGG-1] strain, treated with empty vector (control), *cisd-1(RNAi)*, *ced-9(RNAi)*, *cisd-1/ced-9(RNAi)*, *daf-2(RNAi)* and *unc-51(RNAi)* from hatching. **E** Quantification of the number of GFP::LGG-1 positive puncta per seam cell in DA2123 RNAi treated animals. Values represent mean ± SD (*n* = 35; *P < 0.05, ****P < 0.0001, one-way ANOVA/ Tukey’s multiple comparison test). **F** Representative images of heads, using day-1 adult animals of the HZ589 [p_*sqst-1*_SQST-1::GFP] strain, treated with empty vector (control), *cisd-1(RNAi)*, *ced-9(RNAi)*, *cisd-1/ced-9(RNAi)*, *unc-51(RNAi)* and *bec-1(RNAi)* from hatching. **G** Quantification of the number of SQST-1::GFP positive puncta of HZ589 RNAi treated animals. Values represent mean ± SD (*n* = 30; *P < 0.05, **P < 0.01, ***P < 0.001, one-way ANOVA/ Tukey’s multiple comparison test). Scale bars in all panels are 20 μm.

To gain further insight into the molecular mechanisms mediating the crosstalk between CED-9 and CISD-1, we tested the effects on longevity in *ced-9(n2812)* loss-of-function mutants upon *cisd-1* silencing. We found that, loss of CED-9 function (in *ced-9(n2812)* mutants) fully abrogates the detrimental consequences of *cisd-1* downregulation on survival. Notably, CED-9/CISD-1 double-deficient animals are long-lived, compared to wt (Fig. 4A). We obtained similar results by targeting simultaneously both *cisd-1* and *ced-*9, using a single RNAi vector (Fig. S6A–C).

Next, we sought to characterize the molecular basis of longevity conferred by double CED-9 and CISD-1 deficiency. BCL2, the mammalian ortholog of CED-9, has a dual role in the regulation of apoptosis and autophagy [33, 34]. In addition, CISD2/Miner1, the mammalian ortholog of CISD-1 has been shown to act synergistically with the anti-apoptotic protein BCL2 to inhibit Beclin-1-dependent formation of the omegasome in the ER [18]. Thus, we hypothesized that induction of autophagy underlies the pro-survival effect of combined *cisd-1* and *ced-9* knockdown. To test this hypothesis, we measured the lifespan of CISD-1/CED-9 double deficient animals, upon autophagy inhibition. We found that knockdown of two core autophagy components, *unc-51* (the nematode ATG1/ULK1 ortholog) and *bec-1* (the nematode ATG6/ BECN1 ortholog) fully curtails lifespan extension triggered by loss of both CISD-1 and CED-9 (Fig. 4B, C). These results suggest that inhibition of autophagy impedes the lifespan extension mediated by CISD-1 and CED-9 loss. However, since inhibition of autophagy by either *unc-51(RNAi)* or *bec-1(RNAi)* has an effect on nematode’s lifespan, we cannot exclude the possibility that CISD-1 and CED-9 act independently of the autophagy pathway. In the presence of CED-9 (wt animals), downregulation of *unc-51* further shortens the lifespan of *cisd-1(RNAi)-*treated animals (Fig. S6D). By contrast, *bec-1* downregulation does not further shorten the lifespan of *cisd-1(RNAi)*-treated, wt animals (Fig. S6E). This observation could imply that BEC-1 is required for CISD-1- dependent effects on longevity. In line with this observation, studies in mammalian cells have shown that BCL2 requires CISD2/NAF-1 to inhibit Beclin-1-dependent autophagy [18]. Taken together our findings indicate that induction of autophagy mediates lifespan extension upon depletion of both CISD-1 and CED-9.

To further corroborate this notion, we monitored autophagy induction and autophagic flux, in vivo, by using the DA2123 [p_*lgg-1*_GFP::LGG-1] and the HZ589 [p_*sqst-1*_SQST-1::GFP] reporter strains respectively. LGG-1 is the nematode ortholog for Atg8/LC3, the lipidated form of which decorates autophagosomal membranes. Upon autophagy induction, GFP::LGG-1 forms discrete punctate structures, corresponding to autophagosomes [35]. Although silencing of either *cisd-1* or *ced-9* does not significantly alter autophagosome formation, depletion of both increases the number of autophagosomes in hypodermal seam cells (Fig. 4D, E). The extent of autophagy induction is similar to that observed upon downregulation of *daf-2*, which triggers autophagy by reducing insulin signaling [36]. Nematodes treated with *unc-51(RNAi)* display significantly lower number of GFP::LGG-1 positive puncta, as expected (Fig. 4E). Notably, depletion of both CISD-1 and CED-9 further increases the number of autophagosomes in *daf-2(e1370)* mutant animals (Fig. S6F, G). Single CISD-1 deficiency also augments autophagosomal number in *daf-2(e1370)* mutants, albeit to a lesser extent compared to that of double deficient animals. To examine whether autophagosomes accumulate due to a blockade in autophagic flux, we monitored the degradation of the autophagic substrate SQST-1, the nematode ortholog of p62/sequestosome [37]. The levels of SQST-1::GFP positive puncta remain unaffected in control, *cisd-1(RNAi)*- and *ced-9(RNAi)*- treated worms. However, simultaneous silencing of *cisd-1* and *ced-9* significantly reduces the number of SQST-1::GFP-positive puncta (Fig. 4F–G), suggesting that autophagy is induced under these conditions. Nematodes treated with *bec-1(RNAi)* or *unc-51(RNAi)* exhibit increased number of SQST-1::GFP-positive puncta, as expected (Fig. 4F-G). Combined, these observations indicate that concomitant loss of CISD-1 and CED-9 is sufficient to induce autophagy, and that lifespan extension in double deficient animals is mediated by induction of autophagy.

### CISD-1 promotes healthspan through CED-9

**Fig. 5:**
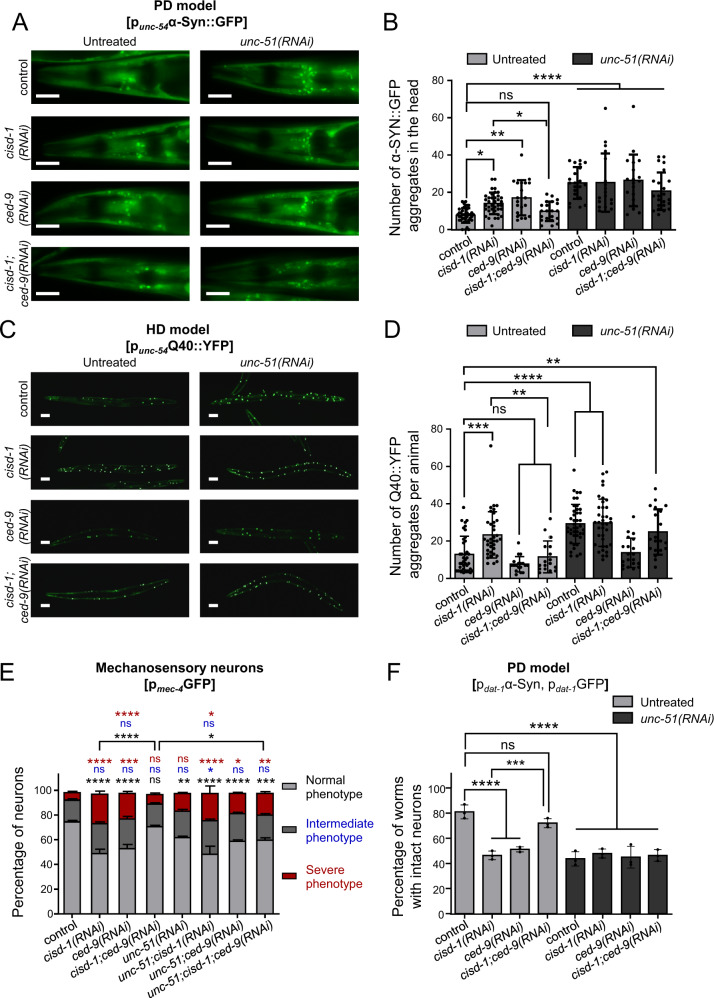
CED-9 is required for proteostasis perturbation and neurodegeneration upon CISD-1 depletion. **A** Representative images of head body wall muscles in day-4 RNAi-treated animals of the PD model strain UA49 [p_*unc-54*_αSyn::GFP] treated with empty vector (control), *cisd-1(RNAi)*, *ced-9(RNAi)*, *cisd-1/ced-9(RNAi)*, *unc-51(RNAi)*, double RNAi for *unc-51* and *cisd-1*, double RNAi for *unc-51* and *ced-9*, double RNAi for *unc-51* and *cisd-1/ced-9* (double RNAi construct). **B** Quantification of α-Syn::GFP positive puncta in head body wall muscles cells of RNAi-treated UA49 animals. Values represent mean ± SD (*n* = 25; ns *P* > 0.05, *P < 0.05, **P < 0.01, ****P < 0.0001, one-way ANOVA/ Tukey’s multiple comparisons test). **C** Representative images of day-1 adult animals of the HD model strain AM141 [p_*unc-54*_Q40::YFP] treated with empty vector (control), *cisd-1(RNAi)*, *ced-9(RNAi)*, *cisd-1/ced-9(RNAi)*, *unc-51(RNAi)*, double RNAi for *unc-51* and *cisd-1*, double RNAi for *unc-51* and *ced-9*, double RNAi for *unc-51* and *cisd-1/ced-9* (double RNAi construct). **D** Quantification of Q40::YFP positive puncta per total animal body in RNAi treated AM141 animals. Values represent mean ± SD (*n* = 35; ns *P* > 0.05, **P < 0.01, ***P < 0.001, ****P < 0.0001, one-way ANOVA/ Tukey’s multiple comparisons test). Scale bars, 20μm. **E** Mechanosensory neurons were observed by using day-4 adult animals of the SK4005 [p_*mec-4*_GFP] reporter strain treated with empty vector (control), *cisd-1(RNAi)*, *ced-9(RNAi)*, *cisd-1/ced-9(RNAi)*, unc-51(RNAi), double RNAi for *unc-51* and *cisd-1*, double RNAi for *unc-51* and *ced-9*, double RNAi for *unc-51* and *cisd-1/ced-9* (double RNAi construct). The neurons were classified into three categories depending on the axonal morphology (normal, intermediate and severe phenotype). The graph represents the percentage of neurons for each category and the statistical differences shown are relative to control RNAi-treated animals. Values represent mean ± SEM from three independent experiments (*n* = 40; ns *P* > 0.05, *P < 0.05, **P < 0.01, ***P < 0.001, ****P < 0.0001, one-way ANOVA/ Tukey’s multiple comparisons test). Scale bars in all panels are 20 μm. **F** Dopaminergic neurons were observed by using day-3 adult animals of the UA196 [*sid-1(pk3321)*;*baIn33*(p_*dat-1*_SID-1, p_*myo-2*_mCherry); (p_*dat-1*_α-Syn, p_*dat-1*_GFP)] reporter strain treated with empty vector (control), *cisd-1(RNAi)*, *ced-9(RNAi)*, *cisd-1/ced-9(RNAi)*, *unc-51(RNAi)*, double RNAi for *unc-51* and *cisd-1*, double RNAi for *unc-51* and *ced-9*, double RNAi for *unc-51* and *cisd-1/ced-9* (double RNAi construct). The graph represents the percentage of intact neurons. Values represent mean ± SEM from three independent experiments (*n* = 20; ns *P* > 0.05, ***P < 0.001 ****P < 0.0001, one-way ANOVA/ Tukey’s multiple comparisons test).

To further dissect the functional interaction between CED-9 and CISD-1, we assessed proteostasis and neurodegeneration in CED-9/CISD-1 double deficient animals. As noted above, aggregation of both α-Syn::GFP and Q40::YFP is exacerbated upon *cisd-1(RNAi)* (Fig. 1H–K). Notably, we find that CED-9 deficiency rescues the *cisd-1(RNAi)*-associated α-Syn and poly-Q aggregation in body wall muscle cells (Fig. 5A–D). In addition, *ced-9(RNAi)* nematodes display elevated α-Syn::GFP aggregates, while their Q40::YFP aggregates remain unchanged, compared to the respective controls. The intrinsic properties of α-Syn and Q40, as well as the age of the worms may account for the observed differences in the aggregation propensity. To examine whether the improved proteostasis upon concurrent CISD-1/CED-9 deficiency is mediated by autophagy, we monitored the number of protein aggregates upon silencing of *unc-51*. In agreement with previous studies [38, 39], the number of aggregates increases upon autophagy inhibition, indicating that autophagy contributes to the elimination of α-Syn and Q40 aggregates in nematodes’ muscles. Moreover, protein aggregation in CISD-1/CED-9 double deficient animals is similar to that in control or CISD-1 deficient worms, upon autophagy inhibition. Thus, deficiency in CED-9 restores proteostasis in CISD-1-depleted animals, by autophagy induction.

In addition to proteostasis, we also assessed susceptibility to neurodegeneration in these animals. We found that degeneration of mechanosensory neurons in CISD-1 deficient nematodes is suppressed upon downregulation of *ced-9* (Fig. 5E). Moreover, a similar restoration was observed in the dopaminergic neurons of CISD-1-deficient animals of the PD-model UA196, upon CED-9 depletion (Fig. 5F). In both cases, suppression of neurodegeneration requires autophagy (Fig. 5E, F). Thus, loss of CED-9 induces autophagy that is required to uphold neuronal integrity, upon CISD-1 depletion.

### Dietary restriction and low insulin signaling mitigate the short lifespan upon CISD-1 deficiency

**Fig. 6:**
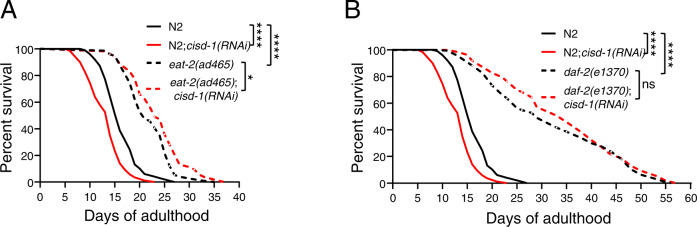
Dietary restriction and low insulin signaling mitigate CISD-1 deficiency. **A** Survival curves of wt (normal solid lines) and *eat-2(ad465)* animals (dashed line) treated with empty vector (black) and *cisd-1(RNAi)* (red) from hatching (*P < 0.05, ****P < 0.0001, Log-rank/Mantel–Cox test). **B** Survival curves of wt (normal solid lines) and *daf-2(e1370)* animals (dashed line) treated with empty vector (black) and *cisd-1(RNAi)* (red) from hatching (ns *P* > 0.05, ****P < 0.0001, Log-rank/Mantel–Cox test).

Attenuation of nutrient sensing signaling pathways, such as the insulin/IGF1, or dietary restriction-related signaling pathways, exert their beneficial effects on ageing by inducing protein quality control mechanisms, such as autophagy [40]. We examined whether dietary restriction or impairment of insulin/IGF1 signaling extend the lifespan of short-lived CISD-1-deficient animals. *eat-2(ad465)* mutant animals display reduced pharyngeal pumping and food uptake, whereas *daf-2(e1370)* mutants are characterized by reduced insulin/IGF signaling. Depletion of CISD-1 in *eat-2(ad465)* mutants significantly extends mean lifespan (Fig. 6A). In addition, the detrimental effect of CISD-1 depletion on lifespan is fully abrogated in *daf-2(e1370)* mutants (Fig. 6B). We conclude that, deterioration of longevity, caused by CISD-1 deficiency, is ameliorated upon disruption of the insulin pathway or by dietary restriction.

### Perturbation of iron homeostasis influences lifespan and healthspan in a CISD-1-dependent manner

**Fig. 7:**
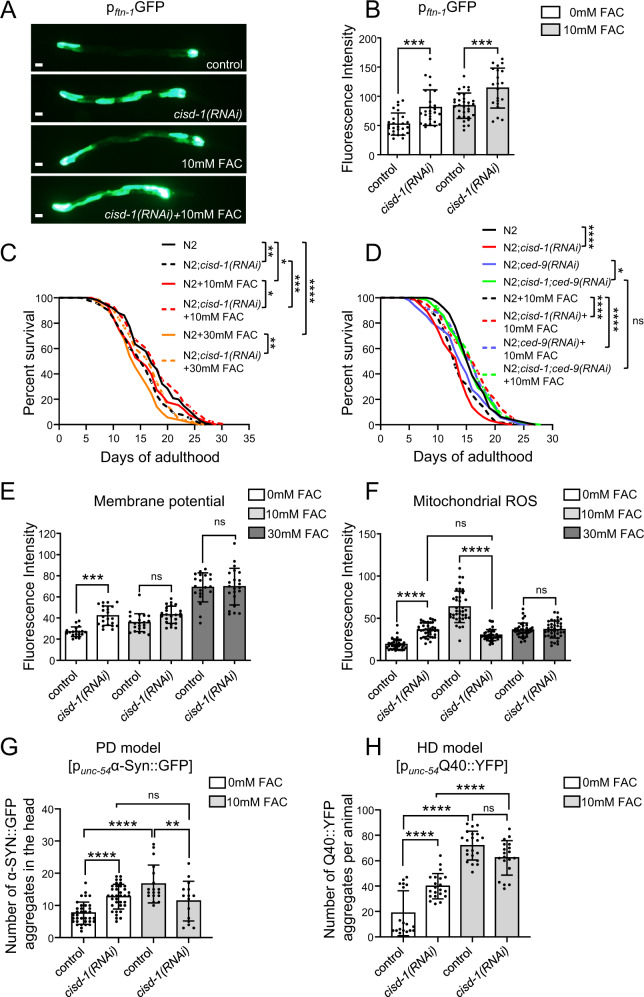
CISD-1 upholds iron homeostasis, which in turn modulates longevity and healthspan. **A** Representative images of day-1 adult animas of the GA631 [p_*ftn-1*_GFP] strain treated with empty vector (control) or *cisd-1(RNAi)* upon normal conditions or mild iron supplementation (10 mM FAC). **B** Quantification of transcriptional *ftn-1* expression by measuring mean fluorescence intensity of RNAi treated GA631 animals. Values represent mean ± SD (*n* = 25; ns *P* > 0.05, ***P < 0.001, one-way ANOVA/ Tukey’s multiple comparison test). Scale bars in all panels are 20 μm. **C** Survival curves of wt animals treated with empty vector (normal solid line) and *cisd-1(RNAi)* (dashed line) under normal conditions (black), mild iron supplementation (10 mM FAC) (red), or high iron supplementation (30 mM FAC) (orange) conditions. The RNAi and iron supplementation treatments were started from hatching (*P < 0.05, **P < 0.01, ***P < 0.001, ****P < 0.0001, Log-rank/Mantel–Cox test). **D** Survival curves of wt animals treated with empty vector (black), *cisd-1(RNAi)* (red), *ced-9(RNAi)* (blue) and *cisd-1/ced-9(RNAi)* (green) under normal conditions (normal line) or mild iron supplementation (10 mM FAC) (dashed line). The RNAi and iron supplementation treatments were started from hatching (ns *P* > 0.05, *P < 0.05, ****P < 0.0001, Log-rank/Mantel–Cox test). **E** Membrane potential was determined by measuring mean fluorescence intensity of day-1 wt animals, treated with either empty vector (control) or *cisd-1(RNAi)* upon normal conditions, mild (10 mM FAC) or high (30 mM FAC) iron supplementation. The animals were supplemented overnight with the selective mitochondrial dye TMRE. Values represent mean ± SD (*n* = 30; ns *P* > 0.05, ***P < 0.001, one-way ANOVA/ Tukey’s multiple comparisons test). **F** For the determination of mitochondrial ROS, mean fluorescence intensity was measured in day-1 wt animals treated with either empty vector (control) or *cisd-1(RNAi)* upon normal conditions, mild (10 mM FAC) or high (30 mM FAC) iron supplementation. The animals were supplemented overnight with the selective mitochondrial dye MitoTracker™ Red CM-H_2_Xros. Values represent mean ± SD (*n* = 50; ns *P* > 0.05, ****P < 0.0001, one-way ANOVA/Tukey’s multiple comparisons test). **G** Quantification of α-Syn::GFP positive puncta in head body wall muscles cells of day-4 animals of the UA49 strain treated with either empty vector (control) or *cisd-1(RNAi)* upon normal conditions, or mild iron supplementation (10 mM FAC). Values represent mean ± SD (*n* = 25; **P < 0.01, ****P < 0.0001, one-way ANOVA/ Tukey’s multiple comparisons test). **H** Quantification of Q40::YFP positive puncta per animal body in day-4 animals of the AM141 strain treated with either empty vector (control) or *cisd-1(RNAi)* upon normal conditions, or mild iron supplementation (10 mM FAC). Values represent mean ± SD (*n* = 25; ns *P* > 0.05, ****P < 0.0001, one-way ANOVA/Tukey’s multiple comparisons test).

Iron is essential for the survival of all organisms. Both iron deficiency and iron overload have detrimental effects on cellular and organismal physiology, highlighting the importance of tightly regulating intracellular iron abundance [41]. Owing to their OMM localization and their ability to bind and release Fe–S clusters, CISD1/mitoNEET and CISD2/NAF-1 proteins have been suggested to play an active role in preserving cellular iron homeostasis [42]. Thus, we investigated the potential role of CISD-1 in iron homeostasis. To this end, we examined whether iron homeostasis is compromised upon CISD-1 deficiency, by monitoring the transcriptional expression of *ftn-1* gene, one of the two ferritin heavy chain *C. elegans* orthologues. FTN-1 is an iron-storage protein, the expression of which is transcriptionally regulated by intracellular iron levels [43, 44]. CISD-1 deficient animals display induced *ftn-1* expression, under baseline conditions and upon iron (Ferric Ammonium Citrate/FAC) supplementation, indicating that cytoplasmic iron concentration is increased and thus iron homeostasis is perturbed (Fig. 7A, B).

We, next, assessed whether CISD-1 effects on longevity and healthspan are due to excessive iron. As previously documented [44, 45], iron supplementation shortens the lifespan of wt animals in a dose-dependent manner (Fig. 7C and Fig. S7). Intriguingly, while *cisd-1(RNAi)*-treated animals are short-lived under normal conditions, iron supplementation significantly extends their lifespan (Fig. 7C). We observed the strongest effect upon mild iron supplementation (10 mM FAC). Combined, these findings indicate that CISD-1 deficiency confers resistance to iron supplementation, and that iron modulates the CISD-1-effects on longevity. Iron supplementation does not alter the lifespan of long-lived, CISD-1/CED-9 double deficient animals, suggesting that iron abundance mediates CED-9-effects on lifespan (Fig. 7D). Mitochondrial membrane potential, as well as ROS levels, which increase upon iron supplementation, are not further altered upon *cisd-1* downregulation, indicating that CISD-1 influences mitochondrial function by impinging on iron homeostasis (Fig. 7E, F). Finally, we examined the link between healthspan and the crosstalk of CISD-1 with iron homeostasis, by monitoring protein aggregation in PD and HD nematode models. Iron supplementation augments α-Syn::GFP and Q40::YFP aggregation in control animals (Fig. 7G, H), suggesting that increased intracellular iron is sufficient to disturb cellular proteostasis, as previously reported [45]. However, iron supplementation does not further increase α-Syn::GFP aggregation in the CISD-1 deficient animals. Additionally, the supplemented CISD-1 deficient animals display reduced α-Syn aggregation compared to the respective controls (Fig. 7G). In the HD model, iron supplementation further increases Q40::YFP aggregation, while the *cisd-1(RNAi)* supplemented animals tend to have reduced number of Q40::YFP aggregates (Fig. 7H). These results suggest that CISD-1 depletion confers resistance to iron proteotoxicity, at least in the PD α-Syn-overexpressing model.

## Discussion

Our study provides insight relevant to the mechanisms through which the OMM iron-sulfur protein CISD-1, the nematode ortholog for both mammalian mitoNEET and CISD2 modulates longevity and healthspan in *C. elegans*. We show that CISD-1 deficiency curtails mean lifespan, induces neurodegeneration and perturbs cellular proteostasis. Notably, we identified conditions under which these negative effects are ameliorated. Thus, *C elegans* may serve as a model for studying Wolfram Syndrome and investigating double NEETs’ deficiency in vivo.

While, we showed that lifespan is significantly affected upon *cisd-1* downregulation, a recent study showed that the lifespan of *cisd-1(tm4993)* null mutants is comparable to that of wt animals [25]. However, after outcrossing the same mutants we did observe a mild but significant lifespan shortening, suggesting that background mutations in the initial strain could mask the effects on longevity. In congruence to longevity, the outcrossed *cisd-1(tm4993)* mutants display defects on neuronal integrity and proteostasis, similar to those observed upon *cisd-1(RNAi)*. Moreover, we found that CISD-dependent effects on longevity depend on mitochondrial activity. In the long-lived mitochondrial mutants *isp-1(qm150)* and *clk-1(e2519)*, the negative effects of *cisd-1* downregulation on longevity are abolished. Our findings indicate that CISD-1 likely sustains mitochondrial bioenergetics. Augmented membrane potential, ATP production and OCR in CISD-1 deficient animals reveal increased mitochondrial function. However, this is accompanied by considerable rise in mitochondrially-derived ROS and concomitant reduction of life expectancy, suggesting that overall organismal homeostasis is compromised. It is still not clear how OMM mitoNEET, or CISD2 protein deficiency impacts mitochondrial function, and there are contradicted reports in the literature [22, 25, 46–50]. In line with our findings, studies in murine models have shown that doxycycline-inducible shRNA-mitoNEET knockdown mice, as well as mouse embryonic fibroblasts (MEFs) deficient for CISD2/Miner1, display significantly higher basal and maximal OCR [22, 47, 51]. While, no increase in mitochondrial mass was observed, ETC was augmented, indicating enhanced energy demands [47].

Notably, loss of the pro-apoptotic CED-3, CED-4, and CED-13, or gain-of-function mutations in the anti-apoptotic CED-9 factor counters the detrimental effect of CISD-1 deficiency on animal lifespan, suggesting that the CED-13-dependent intrinsic apoptosis pathway is required for the pro-longevity effects of CISD-1. The finding that double CED-9 and CISD-1 deficiency extends animal lifespan suggests that, in addition to apoptosis, other pathways contribute to longevity. Indeed, the mammalian CED-9 ortholog, BCL2, is also a negative regulator of autophagy. A study in cancer cell lines has shown that the anti-autophagic role of BCL2 requires direct interaction with CISD2 [18]. Our data show that nematodes deficient for both CISD-1 and CED-9 display extended longevity, reduced neurodegeneration, and improved proteostasis compared to the single CISD-1 deficient animals. The beneficial effects observed upon concurrent loss of CISD-1 and CED-9 require autophagy induction. The observation that single lesion of *ced-9* or *cisd-1* does not induce autophagy implies the existence of alternative CISD-1 or CED-9 interactors for autophagy inhibition. We also showed that dietary restriction (in *eat-2* mutants) or reduced insulin signaling (in *daf-2* mutants), interventions which are known to induce autophagy, are sufficient to reverse the negative effects of CISD-1 deficiency on lifespan. In line with our observations, it has been shown that dietary restriction ameliorates proteostasis and promotes longevity through ER hormesis [52], suggesting that the mitochondrialy-localized CISD-1 may be involved in a similar homeostatic response through mitochondrial-associated membrane (MAM)-related interactions[53, 54].

To obtain further mechanistic insights into how CISD-1, a Fe-S cluster binding protein, mediates its effects, we investigated whether intracellular iron modulates lifespan by interfering with CISD-1 biogenesis and function. Notably, iron supplementation rescues the short lifespan of CISD-1-deficient animals, without interfering with proteostasis. We suggest that the extended lifespan and the general iron resistance of iron supplemented, CISD-1-deficient animals, is mediated by a hormetic response, since these animals are already experiencing oxidative stress, due to increased ROS production.

Lastly, we have identified conditions under which the detrimental effects of CISD-1 deficiency are ameliorated. Specifically, CED-9 deficiency, genetic interventions that trigger autophagy induction, such as dietary restriction and reduced insulin signaling, or mild iron supplementation, are sufficient to decelerate ageing in CISD-1 deficient animals. Moreover, we found that changes in mitochondrial function triggered by iron modulate CISD-1 activity. In turn, CISD-1 modulates longevity by coordinating autophagy and apoptosis. These findings bear relevance to WS2 and could facilitate the development of effective therapeutic interventions for related human pathologies.

## Materials and methods

### Nematode strains and growth conditions

Standard rearing conditions were used for maintaining *C. elegans* strains. All experiments were performed at 20 °C on nematode growth media (NGM) agar supplemented with *Escherichia coli* (OP50 or transformed HT115), unless otherwise indicated. The following nematode strains which are available in CGC were used in the particular study: N2: wild-type Bristol isolate, SK4005: *zdIs5* I [p_*mec-4*_GFP + *lin-15*(+)], AM141: *rmIs133* [p_*unc-54*_Q40::YFP], TK22: *mev-1(kn1)* III, MQ887: *isp-1(qm150)* IV, CB4876: *clk-1(e2519)*, MT4770: *ced-9(n1950)* III, MT7686: *ced-9(n2812)*/ qC1 [*dpy-19(e1259) glp-1(q339)*] III, RB1305: *egl-1(ok1418)* V, MT2547: *ced-4(n1162)* III, MT1522: *ced-3(n717)* IV, MD792: *ced-13(sv32)* X, DA2123: *adIs2122* [p_*lgg-1*_GFP::LGG-1 + *rol-6(su1006)*], MAH14: *daf-2(e1370)* III; *adIs2122* [p_*lgg-1*_GFP::LGG-1 + *rol-6(su1006)*], HZ589: *him-5(e1490)* V; *bpIs151*[p_*sqst-1*_SQST-1::GFP + *unc-76*(+)], GA631: *wuIs177* [p_*ftn-1*_GFP, *lin-15*(+)], SJ4103: *zcls14* [p_*myo-3*_GFP(mit)], SJ4143: *zcIs17* [p_*ges-1*_GFP(mit)], IR28: N2;*Is*[p_*myo-3*_MYO-3::GFP], SJ4058: *zcIs9* [p_*hsp-60*_GFP + *lin-15*(+)], SJ4005: *zcIs4* [p_*hsp-4*_GFP], CL2070: *dvIs70* [p_*hsp-16.2*_GFP + *rol-6(su1006)*], CU1546: *smIs34* [p_*ced-1*_CED-1::GFP + *rol-6(su1006)*]. The initial null *cisd-1(tm4993)* mutant strain was obtained from the National BioResource Project (NBRP) [55].The strains UA196 [*sid-1(pk3321)*;*baIn33* (p_*dat-1*_SID-1, p_*myo-2*_mCherry); (p_*dat-1*_α-Syn, p_*dat-1*_GFP)] and UA49: *baInl2* [p_*unc-54*_α-Syn::GFP + *rol-6*(*su1006*)] were generously provided by Guy Cardwell. The following strains were generated in the current study: IR1571: N2;*Ex*[p_*CEOP2540*_CISD-1::GFP::*unc-54-*3’UTR + *rol-6(su1006)*, IR3111: *cisd-1(tm4993)* 4x outcrossed with the N2 wt strain, IR3140: *cisd-1(tm4993)* 4x;SK4005, IR3123: *cisd-1(tm4993)* 4x;UA49 and IR3122: *cisd-1(tm4993)* 4x; AM141.

### Molecular cloning

For generating the CISD-1::GFP expressing vector, we selected the region between CEOP2540 operon, which contains *cisd-1* gene, and W02B12.11, as a promoter. For the amplification of this particular region we used the primers 5′-AAGCTTTACTTTTTGAGGGGATCAGAA-3′ and 5′-TCTAGAGAAAATCACGAGTAAATTATGTAAA-3′, which include HindIII and XbaI restriction sites respectively. The corresponding product was first inserted in the pCR-II TOPO vector and then in pPD95.77 vector, which had already been digested with HindIII and XbaI. For cloning the *cisd-1* genomic region, we used the primers 5′-TCTAGAATGACCATCGCTGGATTTTGT-3′ and 5′-ACCGGTCCCTTCTTTTCGGATTTGACGATG-3′ for the amplification of *cisd-1* gene. The genomic region was ligated to the pPD95.77 vector, containing CEOP2540 promoter (as described above) by using XbaI and AgeI as restriction sites.

Gene inactivation was achieved by bacterial feeding of *C. elegans* with HT115 bacterial strain transformed with the pL4440 RNAi vector containing the respective gene of interest. For the construction of *cisd-1(RNAi)* plasmid, the following primers were used: 5′- TCTAGAATGCCTTGCCCAACTCAAGT-3’ (FW) (with XbaI restriction site) and 5′-AAACGACCGCTTTGCTTTATT-3′ (RV). The amplified *cisd-1* genomic region was first inserted into the pCRII-TOPO vector and finally ligated into the pL4440 vector upon digestion with XbaI and AgeI. For constructing the *ced-9(RNAi)* plasmid, the following primers were used: 5′-GCTAGCATGACACGCTGCACGGC-3′ (with NheI restriction site) and 5’ AAGCTTAAAAAAACGATTTTTCTGGTTTTTAATG-3′. The amplified *ced-9* genomic region was first inserted into the pCRII-TOPO vector and finally ligated into the pL4440 vector upon digestion with NheI and HindIII. For the double RNAi construct for *cisd-1* and *ced-9, ced-9* genomic region was firstly isolated by digesting the single *ced-9(RNAi)* vector with NheI and HindIII, and subcloned into the *cisd-1(RNAi)* vector, previousy digested with the same enzymes. The *cox-5B* RNAi construct was generated by another lab member by using the primers 5′-GATGGCTCAACTTGCTAAGACG-3′ (FW) and 5′-CATCTCTTTGGATCTCCTTTGC-3′ (RV). *Bec-1* and *unc-51* RNAi constructs were previously generated in our lab [56].

### Nematode strain generation

To generate the IR1571: N2;*Ex*[p_*CEOP2540*_CISD-1::GFP::*unc-54-*3′UTR]; [*rol-6(su1006)*] transgenic animals, we used DNA microinjection in the syncytium region of *C*. *elegans* germlines. Wild-type (N2) young day-1 adult animals were injected with a mix containing the reporter plasmid [p_*CEOP2540*_CISD-1::GFP::*unc-54-*3’UTR] and the selection marker plasmid pRF4[*rol-6(su1006)*], at a final concentration of 25 ng/μl each.

### Lifespan assays

Lifespan assays were performed at 20 °C. Synchronous animal populations were generated by bleaching (hypochlorite treatment) a large amount of gravid adults of the desired strain grown on OP50-seeded NGM plates. Eggs were placed immediately on freshly made RNAi plates, seeded with IPTG-supplemented (2 mM) HT115 bacteria, which had already been transformed with either the empty RNAi vector pL4440 or the indicated RNAi construct. The progeny was grown through the L4 larval stage and then transferred to fresh plates in groups of 20–25 worms per plate for a total of 150–200 individuals per condition. The day after transferring L4 was used as *t* = 1 (days of adulthood). Animals were transferred to freshly made RNAi plates every 2 days until the 12th day of adulthood and every 3 days until the end of the experiment. The animals were examined almost every day and scored as alive or dead (if they do not respond to touch). Nematodes with internally hatched eggs, protruding vulvas, or those which accidentally die during handling or due to dehydration in the edges of the plate, were scored as censored. Each lifespan experiment was repeated at least twice, with one representative experiment shown in the corresponding figures. Statistical analysis was performed using the Log-rank (Mantel–Cox) test of the Prism software package. The statistical analyses of each survival assay are summarized in Supplementary Table 1.

### Analysis of neurodegeneration

Neuronal defects in mechanosensory neurons were assessed by using animals of the SK4005 strain, which had been growing on RNAi for two generations (their hermaphrodite mothers were also grown on RNAi plates from hatching). For each condition 30–40 animals at the fourth day of their adulthood were selected for imaging. The mechanosensory neurons were classified into three categories depending on the axonal morphology: normal phenotype, intermediate phenotype (thin and beaded axons) and severe phenotype (breaks, branches, fork-like structures). For the detection of neuronal defects in dopaminergic neurons as a result of α-Syn aggregation, we used day-3 animals of the dopaminergic RNAi sensitive UA196 strain and determined the percentage of animals which had intact dopaminergic neurons. Each analysis was repeated at least three times. Independent experiments are represented as mean ± SEM in a single graph.

### Analysis of protein aggregation

For the analysis of α-Syn protein aggregation in body wall muscle cells, we used animals of the UA49 strain. 20–30 animals (second generation progeny grown on RNAi) at the fourth day of their adulthood were used for imaging. We imaged only the head region, to consistently measure the same body wall muscles in each worm. For the analysis of poly-glutamine aggregation in body wall muscle cells, we used animals of the AM141 strain. 20–30 day-1 adult animals (second generation progeny grown on RNAi), were used for imaging (at later time points even the control animals had large amount of aggregates, thus the differences between the examined conditions were lost). Q40::YFP aggregates were measured in the whole animal body. In both cases, the animals were collected, anaesthetized with 20 mM tetramisole and mounted on agarose pads for microscopic observation. α-Syn::GFP puncta in the head region, as well as Q40::YFP puncta in the whole animal body, were quantified by using the “Analyze particles” tool of the ImageJ software [57]. Each analysis was repeated at least three times with one representative experiment shown in the corresponding figures.

### Autophagy measurements

Autophagosome number was assessed by using the GFP::LGG-1 reporter strain DA2123, as described in the literature [35]. Approximately 20-25 L4-staged animals (second generation progeny grown on RNAi) were collected, anaesthetized with 20 mM tetramisole and mounted on agarose pads for microscopic observation. The number of the GFP::LGG-1 positive autophagic puncta was quantified in hypodermal seam cells. *daf-2(RNAi)*- and *unc-51(RNAi)*-treated animals were used as positive and negative controls for increased autophagosome number, respectively. We also used the MAH14 strain as an additional control for induced autophagy and similarly measured the number of GFP::LGG-1 positive puncta in hypodermal seam cells. The sequestration of autophagic substrates as an indicator for autophagic flux, was monitored by using the SQST-1::GFP reporter strain (HZ589), as previously described [37]. Approximately 20–30 day-1 adult animals (second generation progeny grown on RNAi), were collected, anaesthetized with 20 mM tetramisole and mounted on agarose pads for microscopic observation. The number of SQST-1::GFP puncta was counted in the head region (just before the beginning of the intestine). Animals treated with *unc-51(RNAi)* and *bec-1(RNAi)* were used as positive controls for increased SQST-1::GFP aggregates.

### Measurements of mitochondrial bioenergetics

For the determination of membrane potential, L4 wt (N2) animals treated with the respective RNAi from hatching were transferred on RNAi plates supplemented with 0.15 μM Tetramethylrhodamine ethyl ester (TMRE). After overnight incubation at 20°, 25–30 animals were mounted in a 20 mM tetramisole drop on microscopic slides, sealed with coverslips and were analyzed in a fluorescence microscope. The same procedure was followed for the determination of mitochondrialy-derived ROS, by using the selective dye MitoTracker™ Red CM-H_2_Xros. For the determination of mitochondrial mass, we used the SJ4143 strain. Day-1 RNAi-treated animals were mounted on slides with tetramisole (in a similar procedure as described above), and were analyzed for the assessment of mean fluorescence intensity in a fluorescence microscope. The determination of ATP production was performed in day-1 RNAi-treated wt (N2) animals, as previously described [58], by using the ATP bioluminescence assay kit CLS II (Roche Diagnostics). Four independent experiments were performed. Approximately 80 animals were used per condition for each experiment. For the measurement of oxygen consumption rate (OCR) in day-1 RNAi treated wt (N2) animals, we followed a previously described method using a Clark-type polarographic oxygen sensor electrode [59]. Three independent experiments were performed, with each experiment including four technical replicates per condition. Approximately 300–400 gravid adults were used per technical replicate. For the normalization of ATP and OCR measurements, total protein content of the respective samples was determined by using the Pierce™ BCA Protein Assay Kit (Thermo Scientific™).

### mRNA quantification

Total RNA from synchronized day-1 animals was extracted by using the TRIzol reagent (Invitrogen). cDNA synthesis was performed by using the iScript™ cDNA Synthesis Kit (Bio-Rad). Quantitative Real Time PCR was performed in a Bio-Rad CFX96 Real-Time PCR system, and was repeated at least three times. The following primer pairs were used in the current study: for measuring *cisd-1* mRNA levels: FW- 5′-GCTCTTATTGGATACCTTGTTGG-3′ and RV-5′-ATGAGTGGGCCAACATTGTC-3′ and for measuring *ced-9* mRNA levels: FW-5′-GATATCGAGGGATTTGTGGTCGA-3′ and RV-5′-CTGTCTGTGCATTTCCAACCGT-3′.

### Monitoring germline apoptosis

Day-1 adult animals of the CU1546 strain [p_*ced-1*_CED-1::GFP], which were RNAi treated from hatching, were used for the determination of germline apoptosis. 25–30 animals per condition were mounted in microscope slides as previously described, and the number of CED-1 GFP positive engulfed cell corpses were scored by observing each gonad under a fluorescence microscope.

### Thrashing assay

Thrashing behavior is typically assessed to identify differences in nematodes’ motility. Day-3 adult wt animals, which were RNAi treated from hatching, were individually placed in a 20 μl drop of M9 buffer on a microscope slide and observed under a stereoscope. The number of thrashes/body bends for each individual worm was counted every time the worm moved in the opposite direction of the previous body bend. The duration of each measurement was 15 s. For each condition, 20 individual animals were used and each one was measured at least three independent times.

### Microscopy and quantification

Worms were immobilized in a 20 mM tetramisole drop on microscopic slides, sealed with coverslips and analyzed with a Zeiss AxioImager Z2 epifluorescence microscope, or EVOS FV Auto 2 Imaging system (Thermofisher Scientific) or Zeiss LSM 710 multiphoton confocal microscope. Quantification of the mean pixel intensity or particle analyses were performed by using the ImageJ software [57].

### Statistical analysis

Statistical analyses were carried out using the Prism software package (version 8; GraphPad Software; https://www.graphpad.com). Data are reported as the mean values ± standard deviation (SD), unless otherwise stated. For statistical analyses, *p* values were calculated by unpaired Student’s t-test and one-way ANOVA with Tukey’s multiple comparisons test. The significance was determined by the *p*-values: *(*p* < 0.05), **(*p* < 0.01), ***(*p* < 0.001), ****(*p* < 0.0001) and n.s. = not significant (*p* > 0.1).

### Reporting summary

Further information on research design is available in the Nature Portfolio Reporting Summary linked to this article.

## Supplementary information

Reporting Summary checklist

Supplementary material

Supplementary material

Supplementary Figure 1

Supplementary Figure 2

Supplementary Figure 3

Supplementary Figure 4

Supplementary Figure 5

Supplementary Figure 6

Supplementary Figure 7

## Data Availability

All data generated or analyzed during this study are available from the corresponding author upon reasonable request.
