# Supplementary material for: Coupling of autophagy and the mitochondrial intrinsic apoptosis pathway modulates proteostasis and ageing in Caenorhabditis elegans
Source: Cell Death Dis. 2023 Feb 11;14(2):110. doi: 10.1038/s41419-023-05638-x (PMC9922313; doi:10.1038/s41419-023-05638-x)
Supplement: Supplementary file 2 — Supplementary material [file 41419_2023_5638_MOESM2_ESM.docx]

**Coupling of autophagy and the mitochondrial intrinsic apoptosis pathway modulates proteostasis and ageing in *Caenorhabditis elegans***

Christina Ploumi, Emmanouil Kyriakakis and Nektarios Tavernarakis


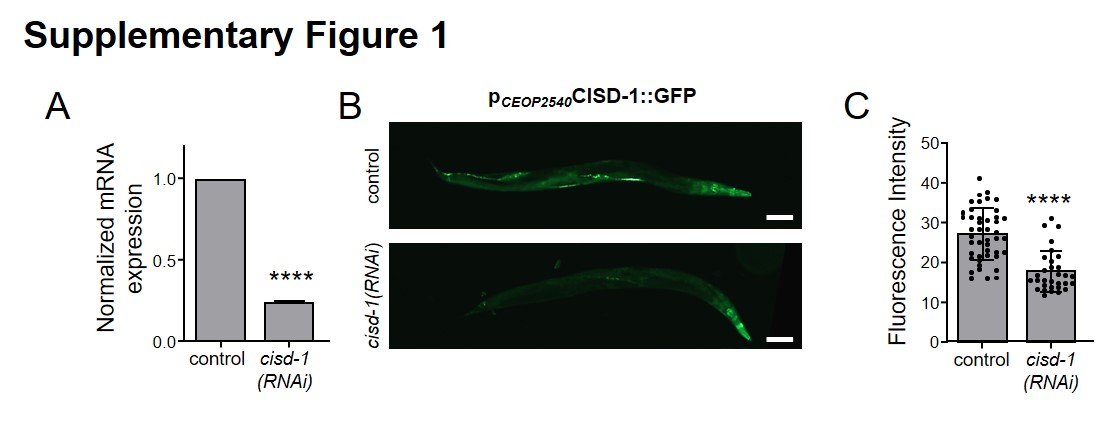
**Supplementary Material**

**Supplementary Figure 1. Efficacy of RNAi-mediated *cisd-1* depletion. A.** *cisd-1* transcription levels in day-1 wt animals treated with empty vector (control) or *cisd-1(RNAi)* from hatching. mRNA expression levels are relative to control and are normalized to the reference gene *pmp-3*. Values represent mean ± SEM (**** *P<0.0001*, unpaired t-test). **B.** Representative images of day-1 p*_CEOP2540_*CISD-1::GFP transgenic animals treated with empty vector (control) or *cisd-1(RNAi)* from hatching. **C.** Quantification of CISD-1 protein expression by measuring mean fluorescent intensity of day-1 p*_CEOP2540_*CISD-1::GFP transgenic animals. Values represent mean ± SD (n=30; **** *P<0.0001*, unpaired t-test). Scale bars in all panels are 20μm.


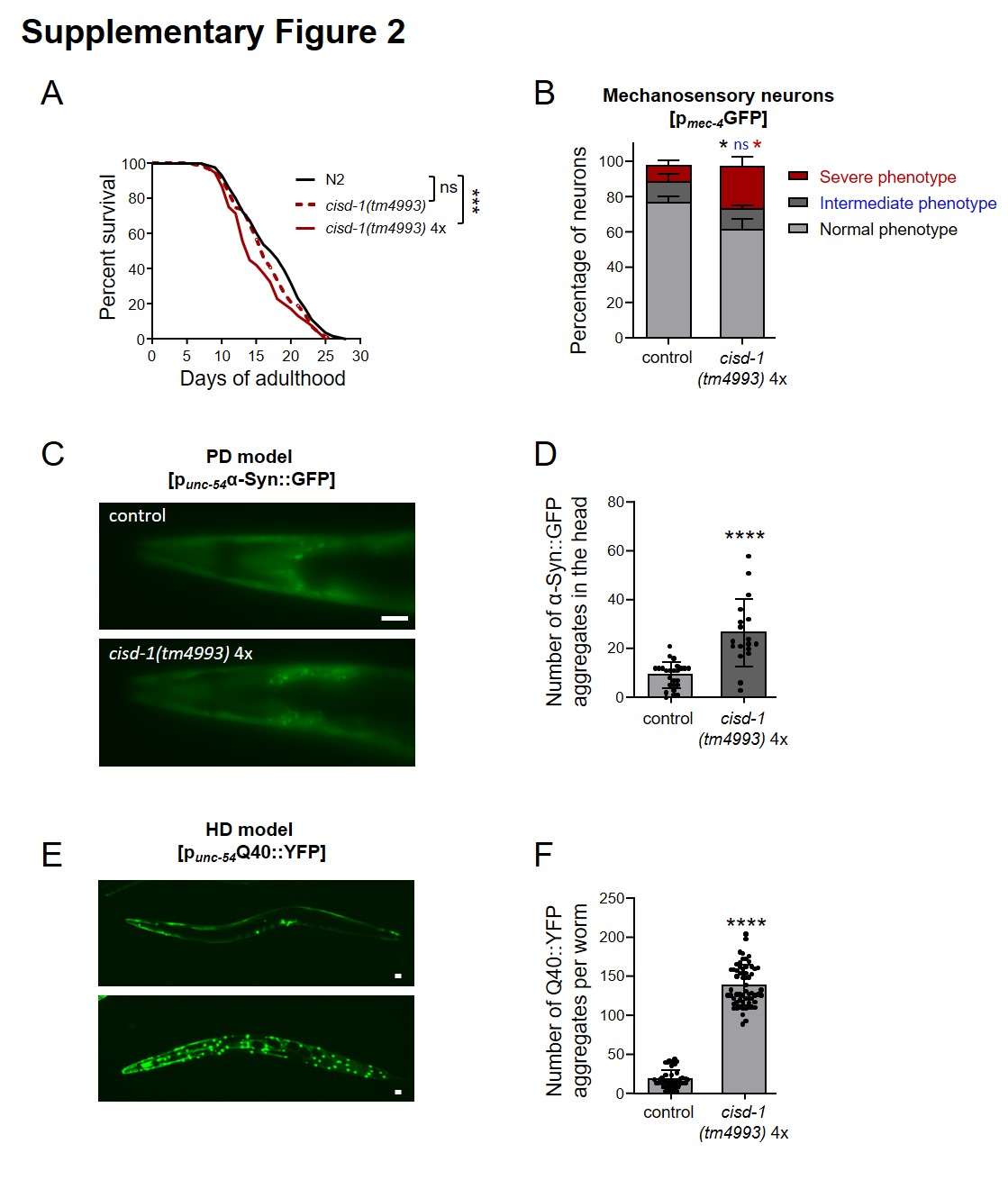


**Supplementary Figure 2. Effects of *cisd-1* ablation on longevity, neuronal integrity and proteostasis. A.** Survival curves of wt (normal black line), *cisd-1(tm4993)* (dashed red line) and the 4x outcrossed *cisd-1(tm4993)* mutants treated with empty vector (ns *P>0.05*, *** *P<0.001,* Log-rank/Mantel-Cox test). **B.** Mechanosensory neurons were observed by using day-4 adult animals of the SK4005 [p*_mec-4_*GFP] and IR3140 [*cisd-1(tm4993)* 4x ;SK4005] strains. The neurons were quantified depending on their axonal morphology (normal, intermediate and severe phenotype). Values represent mean ± SEM from three independent experiments (n=40; *** *P<0,05*, two-way ANOVA/ Sidak’s multiple comparison test). **C.** Representative images of head body wall muscles in day-4 animals of the UA49 [p*_unc-54_*α-Syn::GFP] and IR3123 [*cisd-1(tm4993)* 4x;UA49] strains **D.** Quantification analysis of α-Syn::GFP puncta in body wall muscles of the head region. Values represent mean ± SD (n=25; **** *P<0.0001*, unpaired t-test). **E.** Representative images of day-1 adult animals of the AM141 [p*_unc-54_*Q40::YFP] and IR3122 [*cisd-1(tm4993)* 4x; AM141] strains. **F.** Quantification analysis of Q40::YFP puncta in the entire body. Values represent mean ± SD (n=47; **** *P<0.0001*, unpaired t-test). Scale bars in all panels are 20μm.


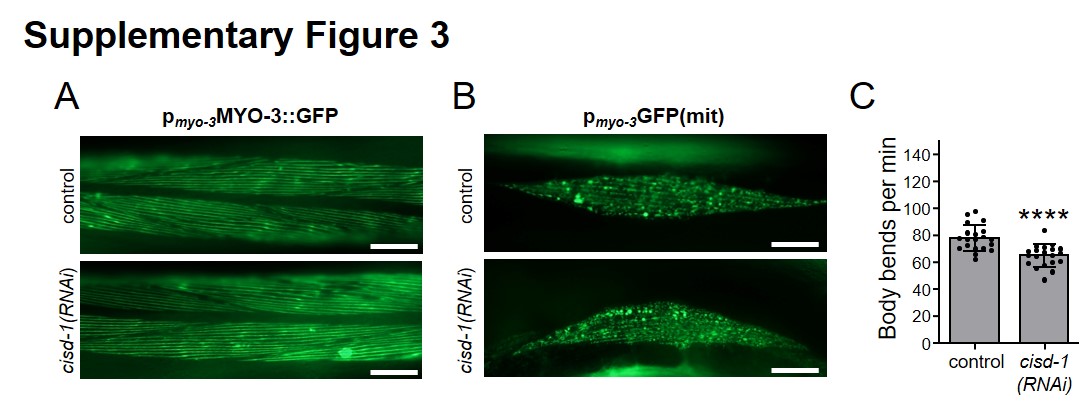


**Supplementary Figure 3. CISD-1 deficiency induces locomotory defects without altering the muscle mitochondrial network or muscular morphology. A.** Representative images of myofilament networks in day-3 animals of the IR28 [p*_myo-3_*MYO-3::GFP] strain treated with empty vector (control) or *cisd-1(RNAi)*. **B.** Representative images of muscle mitochondrial network in day-3 animals of the SJ4103 [p*_myo-3_*GFP(mit)] strain treated with empty vector (control) or *cisd-1(RNAi)*. **C.** Body bending analysis of day-3 wt animals treated with empty vector (control) or *cisd-1(RNAi)*. Values represent mean ± SD (n=20; **** *P<0.0001*, unpaired t-test). Scale bar, 20μm


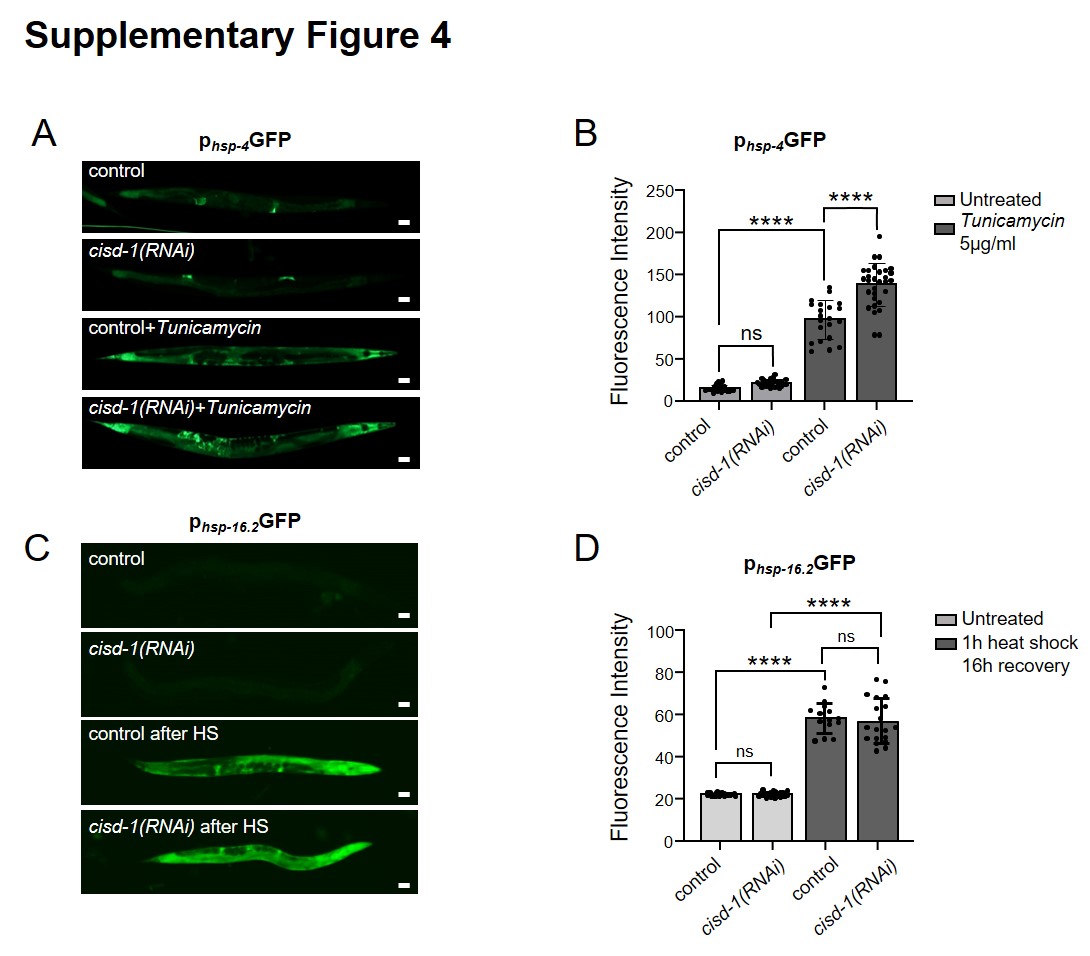


**Supplementary Figure 4. CISD-1 and protein misfolding. A.** Representative images of day-4 animals of the SJ4005 [p*_hsp-4_*GFP] strain treated with empty vector (control) or *cisd-1(RNAi)* upon normal conditions or upon exposure to tunicamycin (5μg/ml, 24h). **B.** Mean fluorescence intensity values of RNAi treated animals of the SJ4005 strain. Values represent mean ± SD (n=25; ns *P>0,05*, **** *P<0.0001*, unpaired t-test). **C.** Representative images of day-4 animals of the CL2070 [p*_hsp-16.2_*GFP] strain treated with empty vector (control) or *cisd-1(RNAi)* upon normal or heatshock conditions (1h at 37°C). **D.** Mean fluorescence intensity values of RNAi treated animals of the CL2070 strain. Values represent mean ± SD (n=25; ns *P>0,05*, ***** P<0.0001*, unpaired t-test). Scale bars in all panels are 20μm.


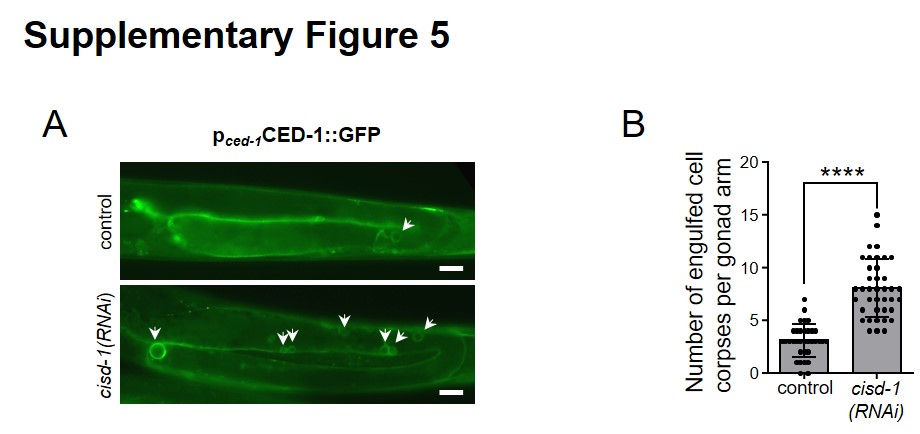


**Supplementary Figure 5. Anti-apoptotic function of CISD-1. A.** Representative images of day-1 animals of the CU1546 [p*_ced-1_*CED-1::GFP] strain, treated with empty vector (control) or *cisd-1(RNAi)*. B. Quantification of engulfed cell corpses in the gonads of RNAi treated animals of the CU1546 strain. Values represent mean ± SD (n=30; **** *P<0.0001*, unpaired t-test). Scale bars in all panels are 20μm.


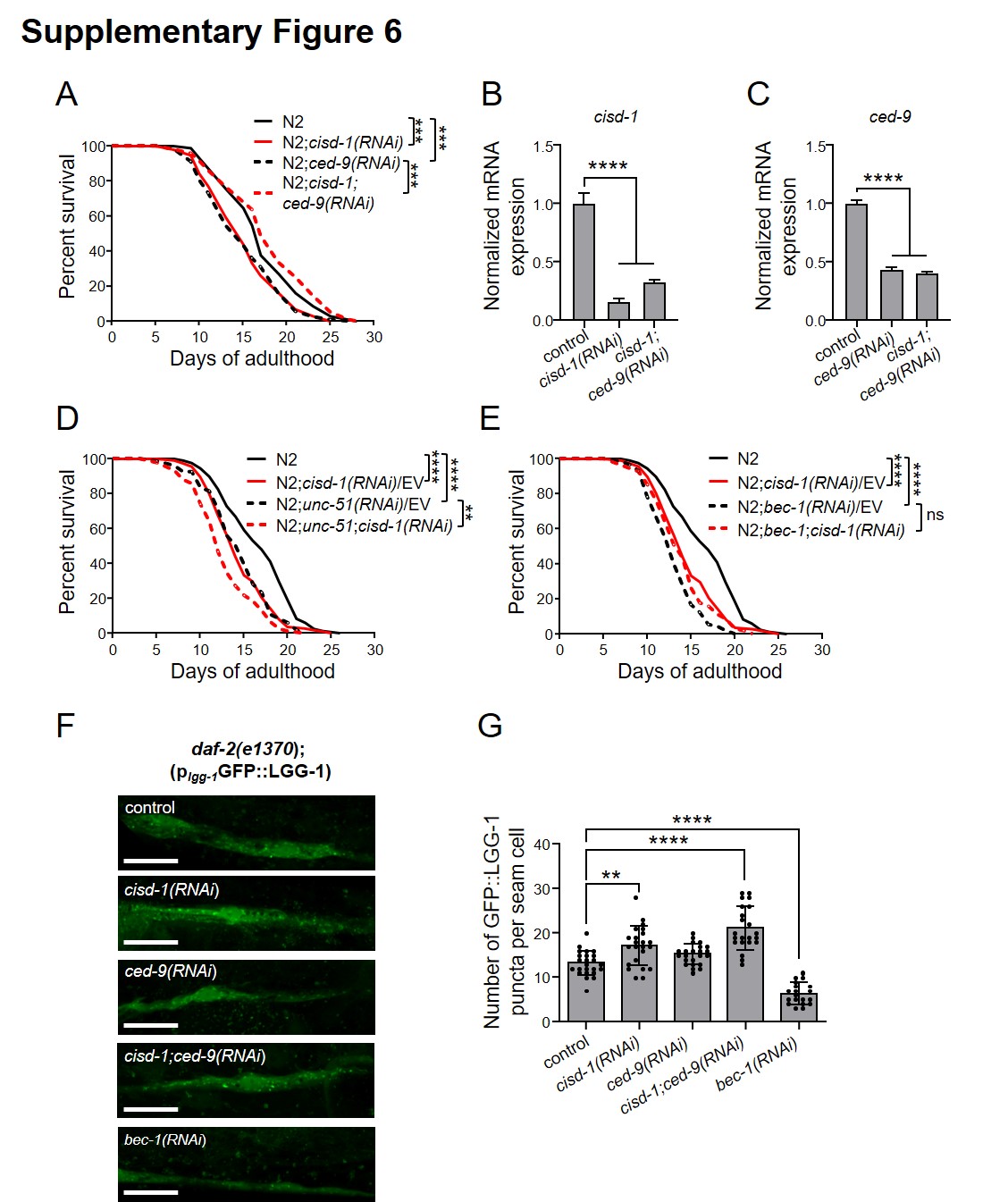


**Supplementary Figure 6. Autophagy mediates epistasis of CED-9 to CISD-1. A.** Survival curves of wt animals treated with empty vector (normal green line), *cisd-1(RNAi)* (normal red line), *ced-9(RNAi)* (green dashed line) and *cisd-1/ced-9(RNAi)* (red dashed line) from hatching (*** *P<0.001,* Log-rank/Mantel-Cox test). **B.** Transcription levels of *cisd-1* gene in day-1 wild-type animals treated with empty vector (control), *cisd-1(RNAi)*, or *cisd-1/ced-9(RNAi)* (double RNAi vector) from hatching. mRNA levels expression were normalized to the reference gene *pmp-3*. Values represent mean ± SEM (**** *P*<0.0001, unpaired t-test). **C.** Transcription levels of *ced-9* gene in day-1 wt animals treated with empty vector (control), *ced-9(RNAi)* or *cisd-1/ced-9(RNAi)* (double RNAi vector) from hatching. mRNA levels expression were normalized to the reference gene *pmp-3*. Values represent mean ± SEM (**** *P*<0.0001, unpaired t-test). **D.** Survival curves of wt animals treated with empty vector (normal green line), *cisd-1(RNAi)* diluted with empty vector (normal red line), *unc-51(RNAi)* diluted with empty vector (green dashed line) and double RNAi for *cisd-1* and *unc-51* genes (red dashed line) from hatching (** *P<0.01*, **** *P<0.0001,* Log-rank/Mantel-Cox test). **E.** Survival curves of wt animals treated with empty vector (normal green line), *cisd-1(RNAi)* diluted with empty vector (normal red line), *bec-1(RNAi)* diluted with empty vector (green dashed line) and double RNAi for *cisd-1* and *bec-1* genes (red dashed line) from hatching (ns *P>0.05*, **** *P<0.0001,* Log-rank/Mantel-Cox test). **F.** Representative images of hypothermal seam cells, using L4-staged animals of the MAH14 [p*_lgg-1_*GFP::LGG-1;*daf-2(e1370)*] strain, treated with empty vector (control), *cisd-1(RNAi)*, *ced-9(RNAi)*, *cisd-1/ced-9(RNAi)* and *bec-1(RNAi)*. **G.** Quantification of GFP::LGG-1 positive puncta per seam cell in RNAi treated animals of the MAH14 strain. Values represent mean ± SD (n=35; *****P<0,0001*, one-way ANOVA/ Tukey’s multiple comparison test). Scale bars in all panels are 20μm.

**
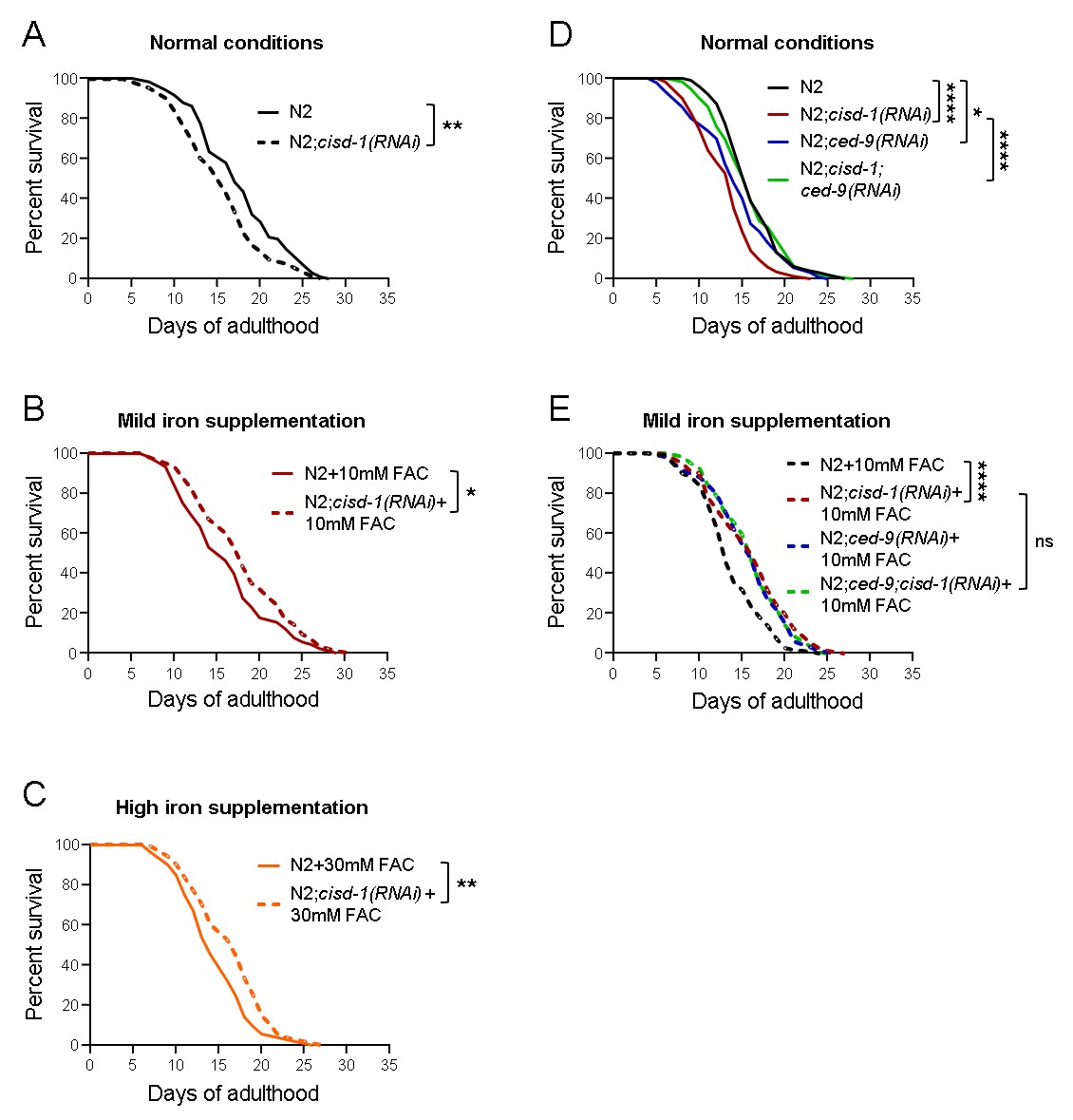
**

**Supplementary Figure 7. Iron abundance is critical for the CISD-1 dependent effects on longevity. A-C.** The survival curves of Fig. 7C are split into three additional graphs. Survival curves of wt animals treated with empty vector (normal solid line) and *cisd-1(RNAi)* (dashed line) under normal conditions (black), mild iron supplementation (10mMFAC) (red), or high iron supplementation (30mM FAC) (orange) conditions. The RNAi and iron supplementation treatments were started from hatching (* *P<0.05*, ** *P<0.01,* Log-rank/Mantel-Cox test). **D-E.** The survival curves of Fig. 7D are split into two additional graphs. Survival curves of wt animals treated with empty vector (black), *cisd-1(RNAi)* (red), *ced-9(RNAi)* (blue) and *cisd-1/ced-9(RNAi)* (green) under normal conditions (normal line) or mild iron supplementation (10mM FAC) (dashed line). The RNAi and iron supplementation treatments were started from hatching (ns *P>0.05*, * *P<0.05*, **** *P<0.0001,* Log-rank/Mantel-Cox test).

**Supplementary Table 1.** **Statistical analysis of survival assays.** Unless otherwise mentioned, all survival assays were performed on plates seeded with HT115(DE3) *E. coli* bacteria transformed with the indicated RNAi plasmid constructs. SEM (standard error of the mean) for each condition is determined by analyzing the median lifespan of all performed replicate survival assays. Total number of deaths and censored animals for each condition is presented for all the performed replicate assays, as well as for the representative experiment used in the corresponding figure. *P* values (of the respective experiment used in each corresponding figure) were calculated using the Log-rank (Mantel-Cox) test, as described in Methods.

| **Corresponding figure** | **Genotype** | **Treatment** | **Median lifespan ±SEM**  (days of adulthood) | **Number of experiments** | **Total Deaths/Censored** | **Deaths/Censored**  (of the representative experiments shown) | ***P* value** |
| --- | --- | --- | --- | --- | --- | --- | --- |
| **Figure 1** | - 1. Wild-type (N2) (Fig. 1C) | EV | 16.86±0.2608 | n=21 | 2621/442 | 170/68 |  |
|  | - 1. Wild-type (N2) [Fig. 1C] | *cisd-1(RNAi)* | 14.73±0.30 | n=16 | 2251/273 | 222/27 | <0.0001  *vs* 1.1 |
| **Figure 2** | 2.1. *mev-1(kn1)* [Fig.2F] | EV | 10.50±0.5 | n=2 | 95/123 | 54/25 |  |
|  | 2.2. *mev-1(kn1)* [Fig.2F] | *cisd-1(RNAi)* | 9.00±1.0 | n=2 | 81/152 | 37/46 | <0.05 *vs* 2.1 |
|  | 2.3. *isp-1(qm150)* [Fig.2G] | EV | 20.50±2.5 | n=2 | 134/31 | 59/10 |  |
|  | 2.4. *isp-1(qm150)* [Fig.2G] | *cisd-1(RNAi)* | 21.00±2.0 | n=2 | 113/48 | 32/16 | ns *vs* 2.3 |
|  | 2.5*. clk-1(e2519)* [Fig.2H] | EV | 19.50±3.5 | n=2 | 152/65 | 91/22 |  |
|  | 2.6*. clk-1(e2519)* [Fig.2H] | *cisd-1(RNAi)* | 23.50±1.5 | n=2 | 169/58 | 113/11 | <0.01 *vs* 2.5 |
| **Figure 3** | 3.1. Wild-type (N2) [Fig. 3B-E] | EV | 16.86±0.2608 | n=21 | 2621/442 | 144/45 |  |
|  | 3.2. Wild-type (N2) [Fig. 3B-E] | *cisd-1(RNAi)* | 14.73±0.30 | n=16 | 2251/273 | 195/15 | <0.0001 *vs* 3.1 |
|  | 3.3. *egl-1(ok1418)* [Fig. 3B] | EV | 17.67±0.88 | n=3 | 355/71 | 146/34 | <0.05 *vs* 3.1 |
|  | 3.4. *egl-1(ok1418)* [Fig. 3B] | *cisd-1(RNAi)* | 15.00±0.58 | n=3 | 253/47 | 83/10 | <0.0001 *vs* 3.3 |
|  | 3.5. *ced-9(n1950)* [Fig. 3C] | EV | 15.50±0.50 | n=2 | 233/26 | 140/24 | <0.01 *vs* 3.1 |
|  | 3.6. *ced-9(n1950)* [Fig. 3C] | *cisd-1(RNAi)* | 15.50±0.50 | n=2 | 188/53 | 112/25 | ns *vs* 3.5 |
|  | 3.7. *ced-4(n1162)* [Fig. 3D] | EV | 13.50±0.50 | n=2 | 319/21 | 171/30 | <0.0001 *vs* 3.1 |
|  | 3.8. *ced-4(n1162)* [Fig. 3D] | *cisd-1(RNAi)* | 13.00±0.00 | n=2 | 473/29 | 174/28 | ns *vs* 3.7 |
|  | 3.9. *ced-3(n717)* [Fig. 3E] | EV | 16.00±0.00 | n=2 | 273/31 | 127/18 | <0.0001 *vs* 3.1 |
|  | 3.10. *ced-3(n717)* [Fig. 3E] | *cisd-1(RNAi)* | 15.50±0.50 | n=2 | 258/58 | 129/22 | ns *vs* 3.9 |
|  | 3.11. Wild-type (N2) [Fig. 3F] | EV | 16.86±0.2608 | n=21 | 2621/442 | 110/33 |  |
|  | 3.12. Wild-type (N2) [Fig. 3F] | *cisd-1(RNAi)* | 14.73±0.30 | n=16 | 2251/273 | 133/7 | <0.0001 *vs* 3.11 |
|  | 3.13. *ced-13(sv32)* [Fig. 3F] | EV | 17.00±1.00 | n=2 | 203/25 | 111/13 | ns *vs* 3.12 |
|  | 3.14. *ced-13(sv32)* [Fig. 3F] | *cisd-1(RNAi)* | 18.00±0.00 | n=2 | 210/26 | 113/15 | ns *vs* 3.13 |
| **Figure 4** | 4.1. Wild-type (N2) [Fig. 4A] | EV | 16.86±0.2608 | n=21 | 2621/442 | 144/45 |  |
|  | 4.2. Wild-type (N2) [Fig. 4A] | *cisd-1(RNAi)* | 14.73±0.30 | n=16 | 2251/273 | 195/15 | <0.0001 *vs* 3.1 |
|  | 4.3. *ced-9(n2812)* [Fig. 4A] | EV | 15.40±0.60 | n=5 | 568/73 | 102/20 | <0.01 *vs* 4.1 |
|  | 4.4. *ced-9(n2812)* [Fig. 4A] | *cisd-1(RNAi)* | 18.33±0.88 | n=3 | 241/56 | 80/11 | <0.0001 *vs* 4.3 |
|  | 4.5. *ced-9(n2812)* [Fig. 4B-C] | EV | 15.40±0.60 | n=5 | 568/73 | 141/24 |  |
|  | 4.6. *ced-9(n2812)* [Fig. 4B-C] | *cisd-1(RNAi)* diluted with EV | 20.50±2.50 | n=2 | 183/72 | 95/46 | <0.0001 *vs* 4.5 |
|  | 4.7. *ced-9(n2812)* [Fig. 4B] | *unc-51(RNAi)* diluted with EV | 14.50±0.50 | n=2 | 219/25 | 122/14 | <0.0001 *vs* 4.5 |
|  | 4.8. *ced-9(n2812)* [Fig. 4B] | *cisd-1(RNAi)*; *unc-51(RNAi)* | 12.50±0.50 | n=2 | 225/34 | 132/15 | ns *vs* 4.7 |
|  | 4.9. *ced-9(n2812)* [Fig. 4C] | *bec-1(RNAi)* diluted with EV | 12.50±0.50 | n=2 | 267/47 | 141/33 | <0.0001 *vs* 4.5 |
|  | 4.10. *ced-9(n2812)* [Fig. 4C] | *cisd-1(RNAi)*; *bec-1(RNAi* | 13.00±1.00 | n=2 | 140/37 | 70/23 | ns *vs* 4.9 |
| **Figure 6** | 5.1. Wild-type (N2) [Fig. 6A-B] | EV | 16.86±0.2608 | n=21 | 2621/442 | 99/8 |  |
|  | 5.2. Wild-type (N2) [Fig. 6A-B] | *cisd-1(RNAi)* | 14.73±0.30 | n=16 | 2251/273 | 92/16 | <0.0001 *vs* 5.1 |
|  | 5.3. *eat-2(ad465)* [Fig. 6A] | EV | 21.00±0.5 | n=2 | 123/60 | 74/0 | <0.0001 *vs* 5.1 |
|  | 5.4. *eat-2(ad465)* [Fig. 6A] | *cisd-1(RNAi)* | 23.00±0.5 | n=2 | 131/48 | 53/0 | <0.05 *vs* 5.3 |
|  | 5.5. *daf-2(e1370)* [Fig. 6B] | EV | 30.00±1.00 | n=3 | 408/77 | 138/25 | <0.0001 *vs* 5.1 |
|  | 5.6. *daf-2(e1370)* [Fig. 6B] | *cisd-1(RNAi)* | 33.00±2.00 | n=3 | 399/59 | 112/24 | ns *vs* 5.5 |
| **Figure 7** | 6.1. Wild-type (N2) [Fig.7C] | EV | 16.86±0.2608 | n=21 | 2621/442 | 107/16 |  |
|  | 6.2. Wild-type (N2) [Fig.7C] | *cisd-1(RNAi)* | 14.73±0.30 | n=16 | 2251/273 | 103/16 | <0.01 *vs* 6.1 |
|  | 6.3. Wild-type (N2) [Fig.7C] | EV+10mM FAC | 15.13±0.67 | n=5 | 683/83 | 104/17 | ns *vs* 6.1 |
|  | 6.4. Wild-type (N2) [Fig.7C] | *cisd-1(RNAi)* +10mM FAC | 17.17±0.40 | n=4 | 484/117 | 95/13 | <0.01 *vs* 6.3 |
|  | 6.5. Wild-type (N2) [Fig.7C] | EV+30mM FAC | 13.86±0.26 | n=6 | 736/78 | 108/10 | <0.0001 *vs* 6.1 |
|  | 6.6. Wild-type (N2) [Fig.7C] | *cisd-1(RNAi)* +30mM FAC | 15.67±0.33 | n=6 | 850/111 | 114/9 | <0.01 *vs* 6.5 |
|  | 6.7. Wild-type (N2) [Fig.7D] | EV | 16.86±0.2608 | n=21 | 2621/442 | 99/8 |  |
|  | 6.8. Wild-type (N2) [Fig.7D] | EV+10mM FAC | 15.13±0.67 | n=5 | 683/83 | 112/16 | <0.0001 *vs* 6.7 |
|  | 6.9. Wild-type (N2) [Fig.7D] | *cisd-1(RNAi)* | 14.73±0.30 | n=16 | 2251/273 | 92/16 | <0.0001 *vs* 6.7 |
|  | 6.10. Wild-type (N2) [Fig.7D] | *cisd-1(RNAi)* +10mM FAC | 17.17±0.40 | n=4 | 484/117 | 112/9 | <0.0001 *vs* 6.8 |
|  | 6.11. Wild-type (N2) [Fig.7D] | *ced-9(RNAi)* | 14.67±0.33 | n=3 | 403/50 | 128/11 | <0.05 *vs* 6.7 |
|  | 6.12. Wild-type (N2) [Fig.7D] | *ced-9(RNAi)*  +10mM FAC | 17.00±1.00 | n=2 | 182/22 | 103/10 | <0.0001 *vs* 6.8 |
|  | 6.13. Wild-type (N2) [Fig.7D] | *cisd-1/ced-9(RNAi)* | 17.33±0.88 | n=3 | 266/66 | 102/10 | ns *vs* 6.7  <0.05 *vs* 6.11 |
|  | 6.14. Wild-type (N2) [Fig.7D] | *cisd-1/ced-9(RNAi)*  +10mM FAC | 17.50±1.50 | n=2 | 165/33 | 113/7 | ns *vs* 6.13 |
| **Supplementary Figure 2** | 7.1. Wild-type (N2) [Suppl. Fig.2A] | EV | 16.86±0.2608 | n=21 | 2621/442 | 112/33 |  |
|  | 7.2. *cisd-1(tm4993)* [Suppl. Fig.2A] | EV | 15.67±0.3333 | n=3 | 349/58 | 178/23 | ns *vs* 7.1 |
|  | 7.3. *cisd-1(tm4993)* 4x [Suppl. Fig.2A] | EV | 13.50±0.2887 | n=4 | 479/65 | 115/28 | <0.01 *vs* 7.1 |
| **Supplementary Figure 6** | 8.1. Wild-type (N2) [Suppl. Fig.5A] | EV | 16.86±0.2608 | n=21 | 2621/442 | 122/34 |  |
|  | 8.2. Wild-type (N2) [Suppl. Fig.5A] | *cisd-1(RNAi)* | 14.73±0.30 | n=16 | 2251/273 | 112/35 | <0.001 *vs* 8.1 |
|  | 8.3. Wild-type (N2) [Suppl. Fig.5A] | *ced-9(RNAi)* | 14.67±0.33 | n=3 | 403/50 | 163/16 | <0.001 *vs* 8.1 |
|  | 8.4. Wild-type (N2) [Suppl. Fig.5A] | *cisd-1/ced-9(RNAi)* | 17.33±0.88 | n=3 | 266/66 | 110/21 | <0.0001 *vs* 8.3 |
|  | 8.5. Wild-type (N2) [Suppl. Fig.5B-C] | EV | 16.86±0.2608 | n=21 | 2621/442 | 112/34 |  |
|  | 8.6. Wild-type (N2) [Suppl. Fig.5D-E] | *cisd-1(RNAi)*  diluted with EV | 14.00±0.58 | n=3 | 383/32 | 113/14 | <0.0001 *vs* 8.5 |
|  | 8.7. Wild-type (N2) [Suppl. Fig.5D] | *unc-51(RNAi)*  diluted with EV | 15.00±0.00 | n=2 | 196/48 | 79/21 | <0.0001 *vs* 8.5 |
|  | 8.8. Wild-type (N2) [Suppl. Fig.5D] | *cisd-1(RNAi)*;*unc-51(RNAi)* | 12.50±0.50 | n=2 | 187/18 | 93/18 | <0.01 *vs* 8.7 |
|  | 8.9. Wild-type (N2) [Suppl. Fig.5E] | *bec-1(RNAi)*  diluted with EV | 13.50±0.50 | n=2 | 223/10 | 114/12 | <0.0001 *vs* 8.5 |
|  | 8.10. Wild-type (N2) [Suppl. Fig.5E] | *cisd-1(RNAi)*;*bec-1(RNAi)* | 13.50±0.50 | n=2 | 225/3 | 108/11 | ns *vs* 8.9 |
| **Supplementary Figure 7** | 9.1. Wild-type (N2) [Suppl. Fig.7A] | EV | 16.86±0.2608 | n=21 | 2621/442 | 107/16 |  |
|  | 9.2. Wild-type (N2) [Suppl. Fig.7A] | *cisd-1(RNAi)* | 14.73±0.30 | n=16 | 2251/273 | 103/16 | <0.01 *vs* 9.1 |
|  | 9.3. Wild-type (N2) [Suppl. Fig.7B] | EV+10mM FAC | 15.13±0.67 | n=5 | 683/83 | 104/17 |  |
|  | 9.4. Wild-type (N2) [Suppl. Fig.7B] | *cisd-1(RNAi)* +10mM FAC | 17.17±0.40 | n=4 | 484/117 | 95/13 | <0.01 *vs* 9.3 |
|  | 9.5. Wild-type (N2) [Suppl. Fig.7C] | EV+30mM FAC | 13.86±0.26 | n=6 | 736/78 | 108/10 |  |
|  | 9.6. Wild-type (N2) [Suppl. Fig.7C] | *cisd-1(RNAi)* +30mM FAC | 15.67±0.33 | n=6 | 850/111 | 114/9 | <0.01 *vs* 9.5 |
|  | 9.7. Wild-type (N2) [Suppl. Fig.7D] | EV | 16.86±0.2608 | n=21 | 2621/442 | 99/8 |  |
|  | 9.8. Wild-type (N2) [Suppl. Fig.7D] | *cisd-1(RNAi)* | 14.73±0.30 | n=16 | 2251/273 | 92/16 | <0.0001 *vs* 9.7 |
|  | 9.9. Wild-type (N2) [Suppl. Fig.7D] | *ced-9(RNAi)* | 14.67±0.33 | n=3 | 403/50 | 128/11 | <0.05 *vs* 9.7 |
|  | 9.10. Wild-type (N2) [Suppl. Fig.7D] | *cisd-1/ced-9(RNAi)* | 17.33±0.88 | n=3 | 266/66 | 102/10 | ns *vs* 9.7,  <0.05 *vs* 9.9, <0.0001 *vs* 9.8 |
|  | 9.11. Wild-type (N2) [Suppl. Fig.7E] | EV+10mM FAC | 15.13±0.67 | n=5 | 683/83 | 112/16 | <0.0001 *vs* 9.7 |
|  | 9.12. Wild-type (N2) [Suppl. Fig.7E] | *cisd-1(RNAi)* +10mM FAC | 17.17±0.40 | n=4 | 484/117 | 112/9 | <0.0001 *vs* 9.11 |
|  | 9.13. Wild-type (N2) [Suppl. Fig.7E] | *ced-9(RNAi)*  +10mM FAC | 17.00±1.00 | n=2 | 182/22 | 103/10 | <0.0001 *vs* 9.11 |
|  | 9.14. Wild-type (N2) [Suppl. Fig.7E] | *cisd-1/ced-9(RNAi)*  +10mM FAC | 17.50±1.50 | n=2 | 165/33 | 113/7 | ns *vs* 9.12 |
