# Supplementary material for: Coupling of autophagy and the mitochondrial intrinsic apoptosis pathway modulates proteostasis and ageing in Caenorhabditis elegans
Source: Cell Death Dis. 2023 Feb 11;14(2):110. doi: 10.1038/s41419-023-05638-x (PMC9922313; doi:10.1038/s41419-023-05638-x)
Supplement: Supplementary file 4 — Supplementary Figure 1 [file 41419_2023_5638_MOESM4_ESM.pptx]

## Slide 1
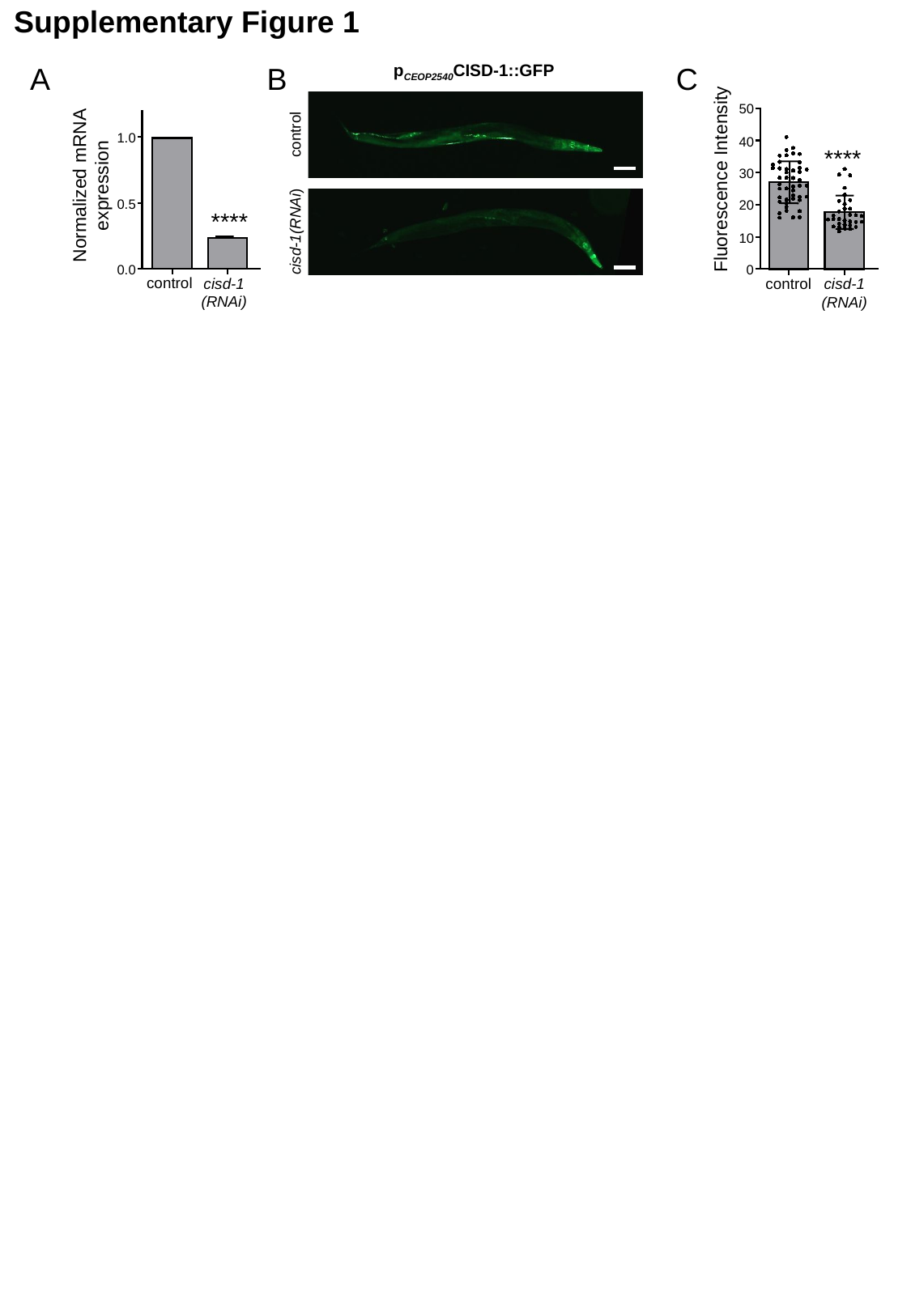

Supplementary Figure 1
A
B
C
50
40
****
30
20
10
0
cisd-1
(RNAi)
control
Fluorescence Intensity
control
1.0
Normalized mRNA
expression
0.5
****
cisd-1(RNAi)
0.0
cisd-1
(RNAi)
control
pCEOP2540CISD-1::GFP
