# Supplementary material for: Coupling of autophagy and the mitochondrial intrinsic apoptosis pathway modulates proteostasis and ageing in Caenorhabditis elegans
Source: Cell Death Dis. 2023 Feb 11;14(2):110. doi: 10.1038/s41419-023-05638-x (PMC9922313; doi:10.1038/s41419-023-05638-x)
Supplement: Supplementary file 5 — Supplementary Figure 2 [file 41419_2023_5638_MOESM5_ESM.pptx]

## Slide 1
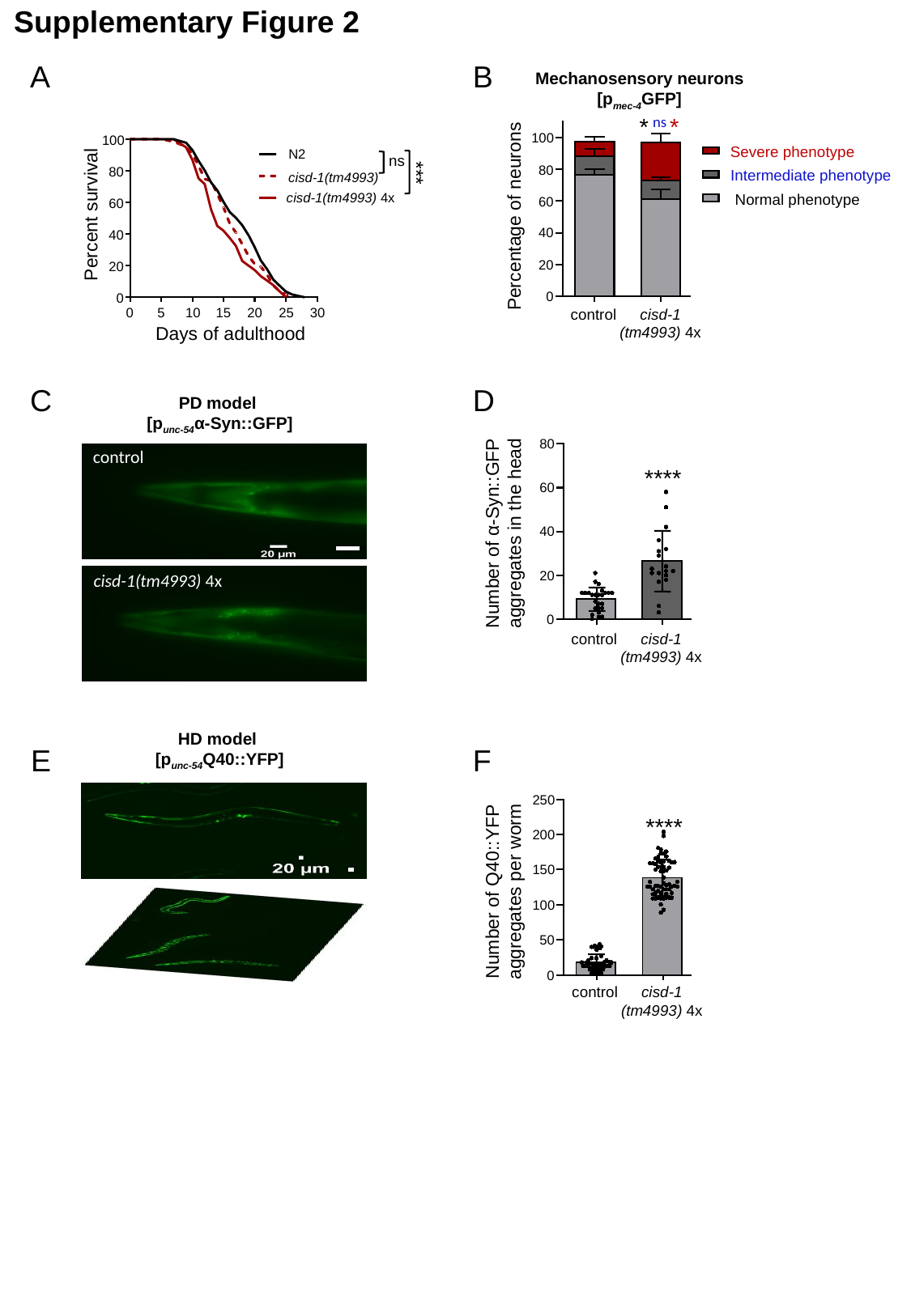

Supplementary Figure 2
A
B
Mechanosensory neurons
[pmec-4GFP]
100
Severe phenotype
80
Intermediate phenotype
Normal phenotype
60
40
20
0
*
ns
*
100
N2
80
cisd-1(tm4993)
cisd-1(tm4993) 4x
60
40
20
0
0
5
10
15
20
25
30
ns
***
Percent survival
Percentage of neurons
control
cisd-1
(tm4993) 4x
Days of adulthood
C
D
PD model
[punc-54α-Syn::GFP]
80
****
60
Number of α-Syn::GFP
aggregates in the head
40
20
0
control
cisd-1
(tm4993) 4x
control
cisd-1(tm4993) 4x
HD model
[punc-54Q40::YFP]
E
F
250
****
200
150
Number of Q40::YFP
aggregates per worm
100
50
0
control
cisd-1
(tm4993) 4x
