# Supplementary material for: Coupling of autophagy and the mitochondrial intrinsic apoptosis pathway modulates proteostasis and ageing in Caenorhabditis elegans
Source: Cell Death Dis. 2023 Feb 11;14(2):110. doi: 10.1038/s41419-023-05638-x (PMC9922313; doi:10.1038/s41419-023-05638-x)
Supplement: Supplementary file 6 — Supplementary Figure 3 [file 41419_2023_5638_MOESM6_ESM.pptx]

## Slide 1
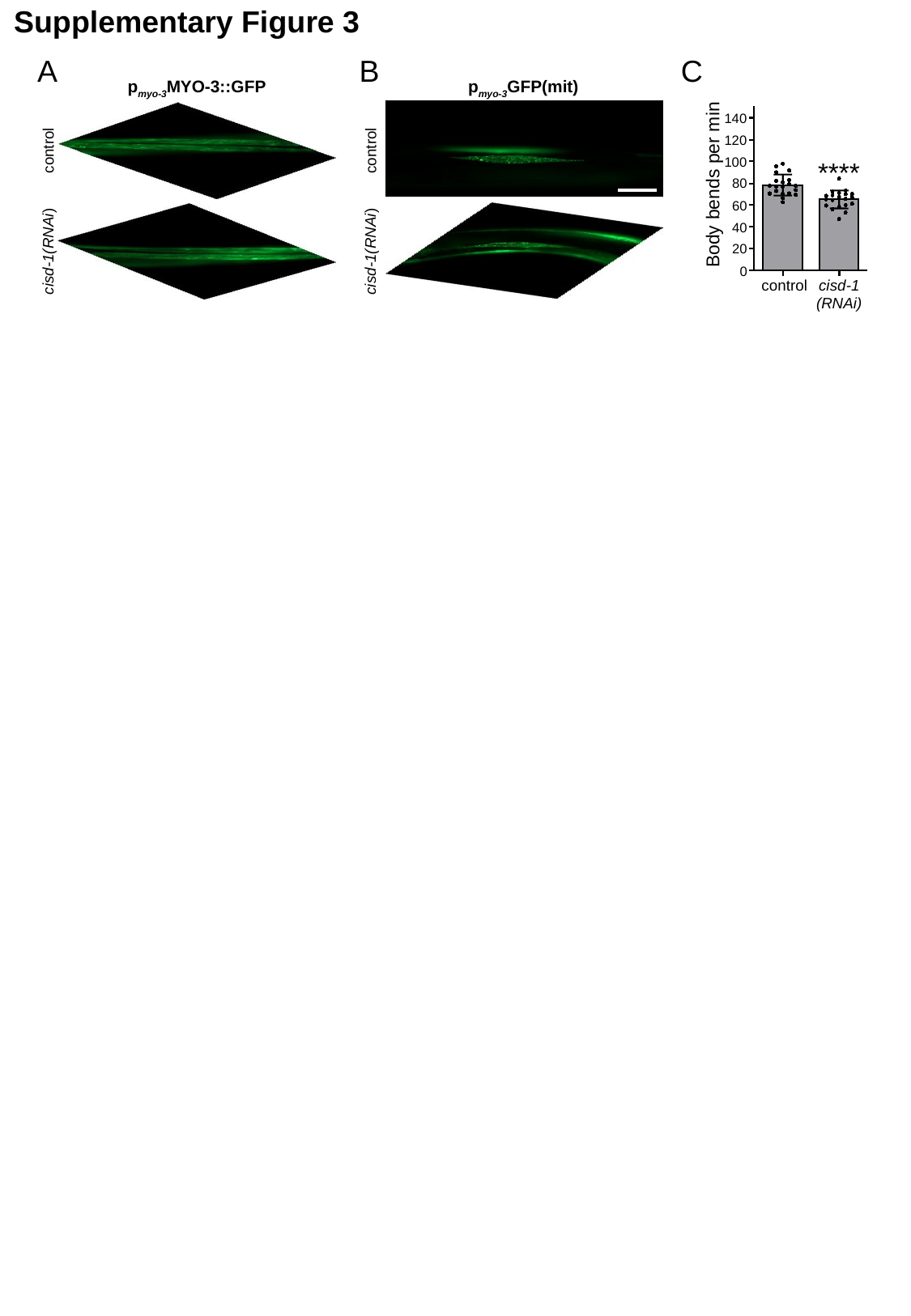

Supplementary Figure 3
A
B
C
pmyo-3MYO-3::GFP
pmyo-3GFP(mit)
140
120
100
****
Body bends per min
80
60
40
20
0
control
cisd-1
(RNAi)
control
control
cisd-1(RNAi)
cisd-1(RNAi)
