# Supplementary material for: Coupling of autophagy and the mitochondrial intrinsic apoptosis pathway modulates proteostasis and ageing in Caenorhabditis elegans
Source: Cell Death Dis. 2023 Feb 11;14(2):110. doi: 10.1038/s41419-023-05638-x (PMC9922313; doi:10.1038/s41419-023-05638-x)
Supplement: Supplementary file 7 — Supplementary Figure 4 [file 41419_2023_5638_MOESM7_ESM.pptx]

## Slide 1
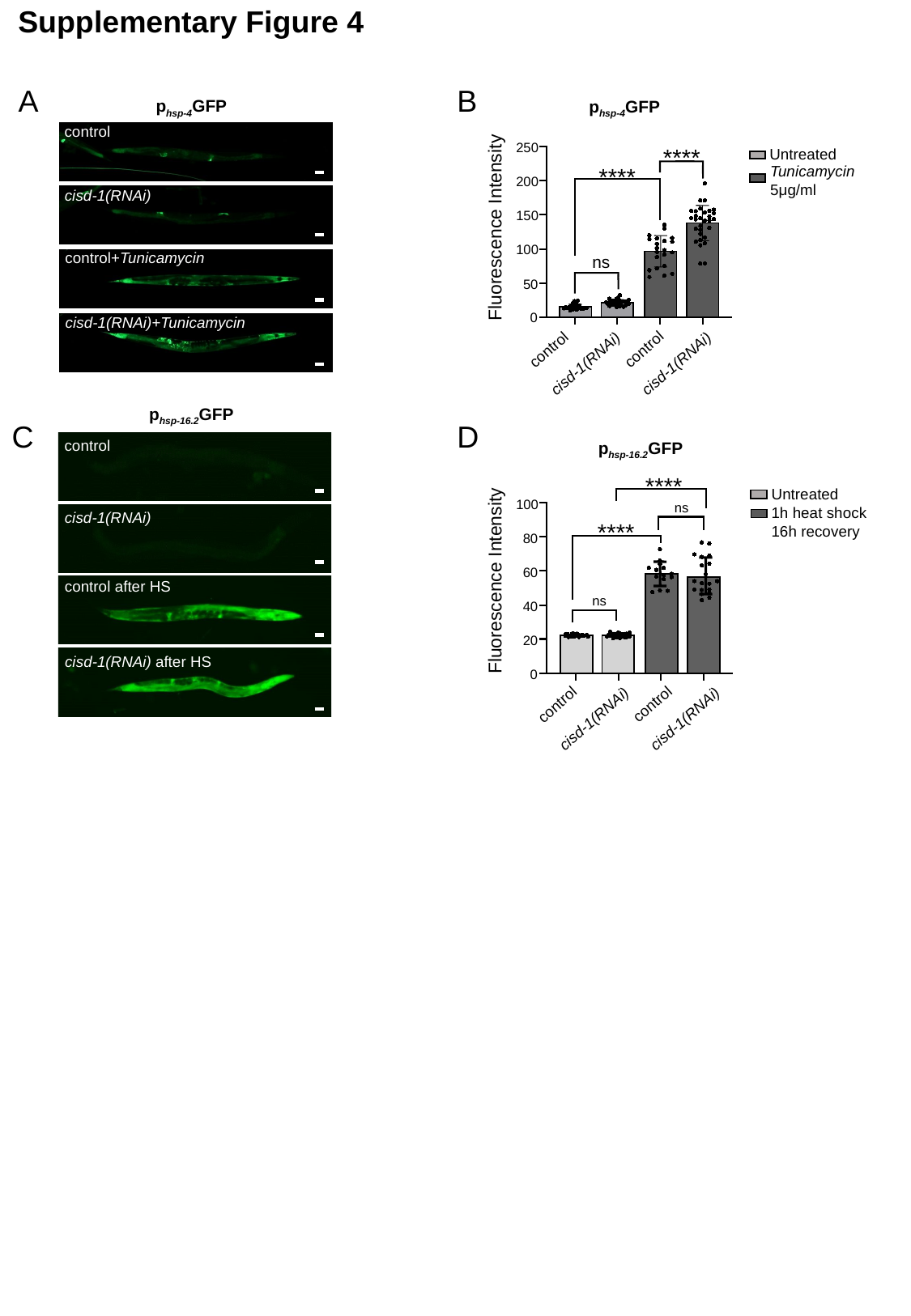

Supplementary Figure 4
A
B
phsp-4GFP
phsp-4GFP
control
250
****
****
200
150
100
ns
50
0
control
control
cisd-1(RNAi)
cisd-1(RNAi)
Untreated
Tunicamycin
5μg/ml
cisd-1(RNAi)
Fluorescence Intensity
control+Tunicamycin
cisd-1(RNAi)+Tunicamycin
phsp-16.2GFP
C
D
control
phsp-16.2GFP
****
100
ns
****
80
60
ns
40
20
0
Untreated
1h heat shock
16h recovery
cisd-1(RNAi)
Fluorescence Intensity
control after HS
cisd-1(RNAi) after HS
control
control
cisd-1(RNAi)
cisd-1(RNAi)
