# Supplementary material for: Coupling of autophagy and the mitochondrial intrinsic apoptosis pathway modulates proteostasis and ageing in Caenorhabditis elegans
Source: Cell Death Dis. 2023 Feb 11;14(2):110. doi: 10.1038/s41419-023-05638-x (PMC9922313; doi:10.1038/s41419-023-05638-x)
Supplement: Supplementary file 8 — Supplementary Figure 5 [file 41419_2023_5638_MOESM8_ESM.pptx]

## Slide 1
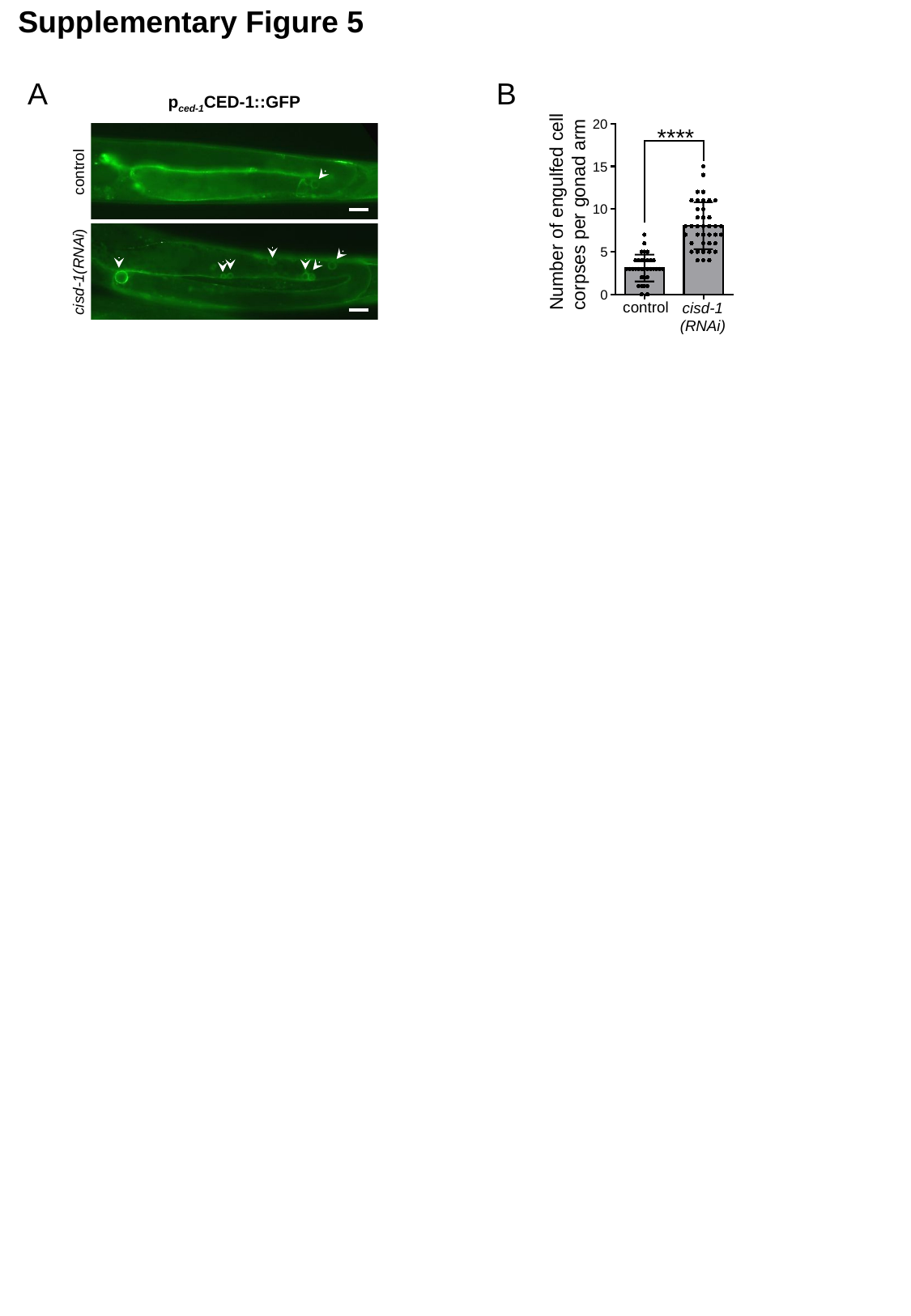

Supplementary Figure 5
A
B
pced-1CED-1::GFP
20
15
10
5
0
****
Number of engulfed cell
corpses per gonad arm
control
cisd-1
(RNAi)
control
cisd-1(RNAi)
