# Supplementary material for: Coupling of autophagy and the mitochondrial intrinsic apoptosis pathway modulates proteostasis and ageing in Caenorhabditis elegans
Source: Cell Death Dis. 2023 Feb 11;14(2):110. doi: 10.1038/s41419-023-05638-x (PMC9922313; doi:10.1038/s41419-023-05638-x)
Supplement: Supplementary file 9 — Supplementary Figure 6 [file 41419_2023_5638_MOESM9_ESM.pptx]

## Slide 1
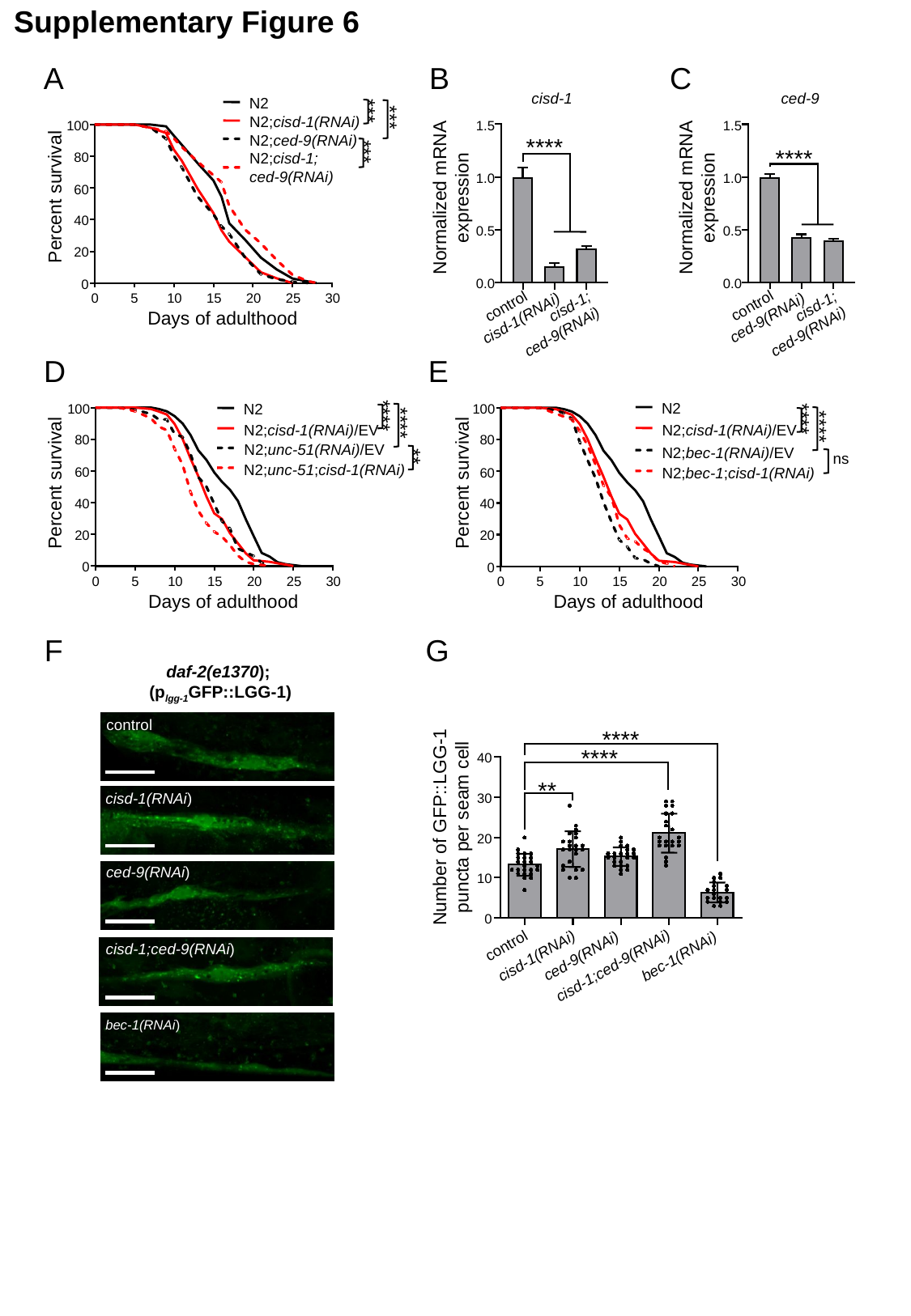

Supplementary Figure 6
A
B
C
cisd-1
ced-9
***
N2
N2;cisd-1(RNAi)
100
N2;ced-9(RNAi)
80
N2;cisd-1;
ced-9(RNAi)
60
40
20
0
0
5
10
15
20
25
30
Days of adulthood
***
1.5
1.5
****
***
****
Normalized mRNA
expression
Normalized mRNA
expression
1.0
1.0
Percent survival
0.5
0.5
0.0
0.0
control
control
cisd-1;
ced-9(RNAi)
cisd-1;
ced-9(RNAi)
ced-9(RNAi)
cisd-1(RNAi)
D
E
N2
100
N2;cisd-1(RNAi)/EV
80
N2;bec-1(RNAi)/EV
N2;bec-1;cisd-1(RNAi)
60
40
20
0
0
5
10
15
20
25
30
Days of adulthood
****
N2
100
****
****
****
N2;cisd-1(RNAi)/EV
80
**
N2;unc-51(RNAi)/EV
ns
N2;unc-51;cisd-1(RNAi)
60
Percent survival
Percent survival
40
20
0
0
5
10
15
20
25
30
Days of adulthood
F
G
daf-2(e1370);
(plgg-1GFP::LGG-1)
control
****
****
40
**
30
20
10
0
cisd-1(RNAi)
Number of GFP::LGG-1
puncta per seam cell
ced-9(RNAi)
control
cisd-1;ced-9(RNAi)
ced-9(RNAi)
cisd-1(RNAi)
bec-1(RNAi)
cisd-1;ced-9(RNAi)
bec-1(RNAi)
