# Supplementary material for: Coupling of autophagy and the mitochondrial intrinsic apoptosis pathway modulates proteostasis and ageing in Caenorhabditis elegans
Source: Cell Death Dis. 2023 Feb 11;14(2):110. doi: 10.1038/s41419-023-05638-x (PMC9922313; doi:10.1038/s41419-023-05638-x)
Supplement: Supplementary file 10 — Supplementary Figure 7 [file 41419_2023_5638_MOESM10_ESM.pptx]

## Slide 1
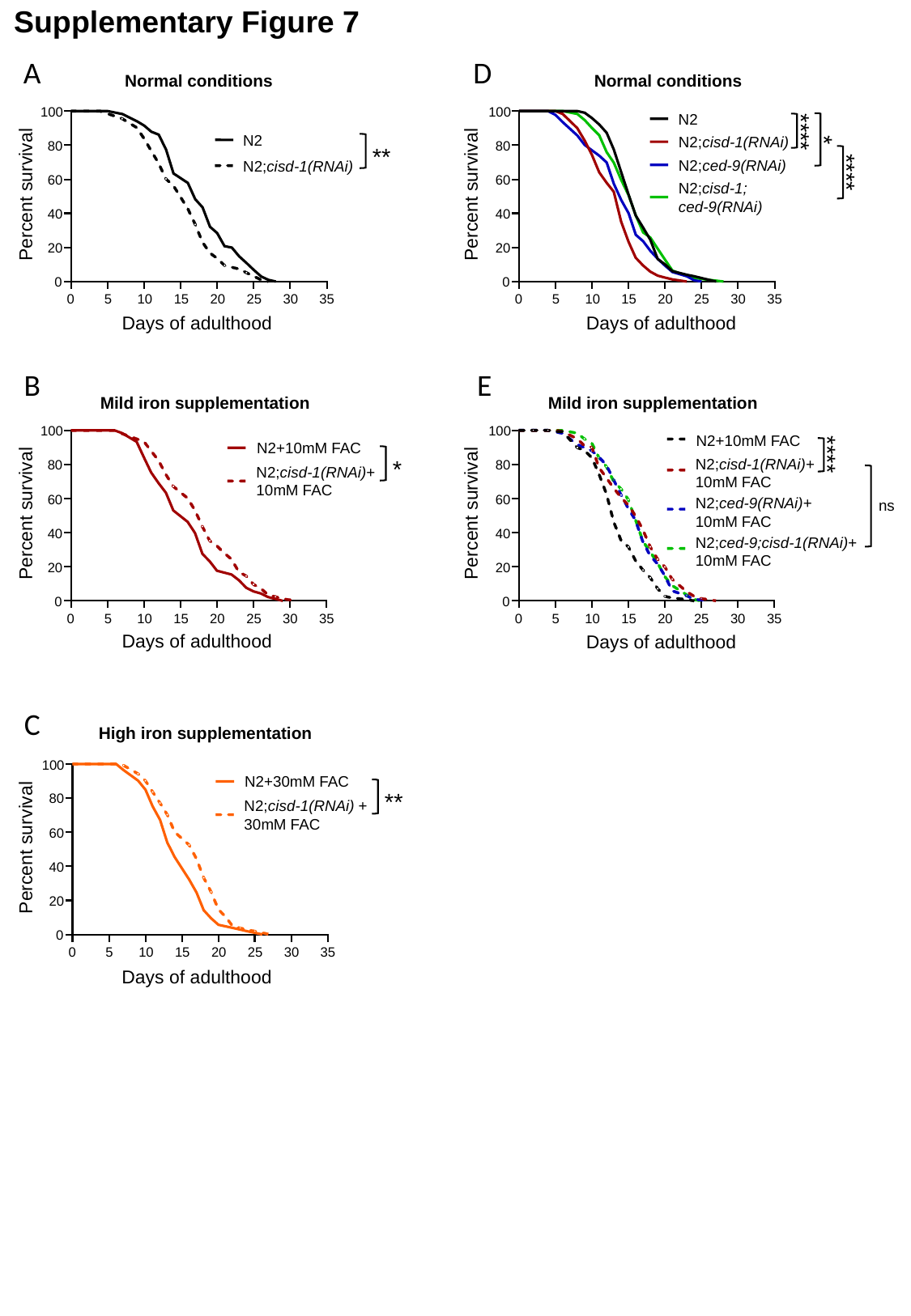

Supplementary Figure 7
A
D
Normal conditions
Normal conditions
100
100
N2
****
*
N2
N2;cisd-1(RNAi)
80
80
**
N2;ced-9(RNAi)
N2;cisd-1(RNAi)
****
60
60
Percent survival
Percent survival
N2;cisd-1;
ced-9(RNAi)
40
40
20
20
0
0
0
5
10
15
20
25
30
35
0
5
10
15
20
25
30
35
Days of adulthood
Days of adulthood
B
E
Mild iron supplementation
Mild iron supplementation
100
100
N2+10mM FAC
N2+10mM FAC
****
*
N2;cisd-1(RNAi)+
10mM FAC
80
80
N2;cisd-1(RNAi)+
10mM FAC
ns
60
60
N2;ced-9(RNAi)+
10mM FAC
Percent survival
Percent survival
40
40
N2;ced-9;cisd-1(RNAi)+
10mM FAC
20
20
0
0
0
5
10
15
20
25
30
35
0
5
10
15
20
25
30
35
Days of adulthood
Days of adulthood
C
High iron supplementation
100
N2+30mM FAC
**
80
N2;cisd-1(RNAi) +
30mM FAC
60
Percent survival
40
20
0
0
5
10
15
20
25
30
35
Days of adulthood
